# Combinatorial Synthesis of Protein–Polymer Conjugates by Postpolymerization Modification of Poly(pentafluorophenyl acrylate)s

**DOI:** 10.1021/acsomega.5c07215

**Published:** 2026-01-08

**Authors:** Emily W. Kish, Thatcher M. Lee, Alexis M. Ziemba, Ama Boamah, Bianca Figueroa, Margherita Piccardi, Carey E. Dougan, Sarah J. Moore, Maren E. Buck

**Affiliations:** 1 Department of Chemistry, 6089 Smith College, Northampton, Massachusetts 01063,United States; 2 Neuroscience Program, 6089 Smith College, Northampton, Massachusetts 01063, United States; 3 Department of Biology, 9310 University of Pisa, Pisa 56126, Italy; 4 Picker Engineering Program, 6089 Smith College Northampton, Massachusetts 01063, United States

## Abstract

Combinatorial methods for preparing polymeric biomaterials enable the rapid identification of materials useful for many applications in science, medicine, and engineering. In the work described here, we demonstrate that side-chain reactive polymers can be used as templates for the rapid preparation of a small library of diversely functionalized protein–polymer conjugates. The activated ester polymer poly­(pentafluorophenyl acrylate) (PPFPA) was modified postpolymerization with substoichiometric equivalents of three hydrophilic primary amines to yield a library of amphiphilic, side-chain reactive copolymers. These copolymers were then conjugated to two receptor-targeting proteins, holotransferrin (hTF) and an engineered fibronectin-based protein (Fn3), through amine-activated ester coupling. We investigated the influence of polymer:protein ratio, side-chain chemistry (i.e., hydrophilic group identity and number of protein-reactive groups), and protein identity on conjugation efficiencies. Our results demonstrate that, for polymers of similar solubility in aqueous media, a larger polymer:protein ratio yields higher conjugation efficiencies. In addition, polymers with a greater number of reactive groups or shorter hydrophilic side chains improve protein conjugation efficiency. Finally, smaller proteins couple to the polymers more efficiently than do larger proteins. Collectively, the results described here demonstrate a modular approach for efficiently preparing bioconjugates with diverse chemistries that may be of interest in a broad range of applications.

## Graphical abstract

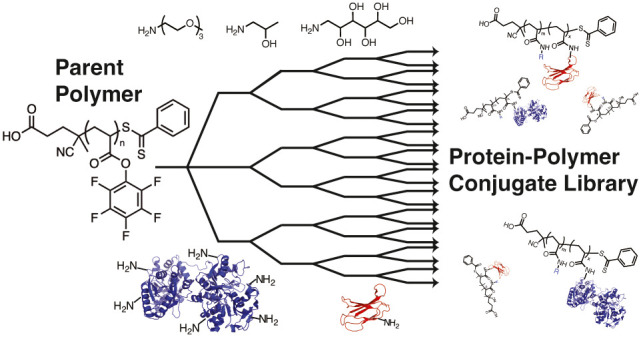

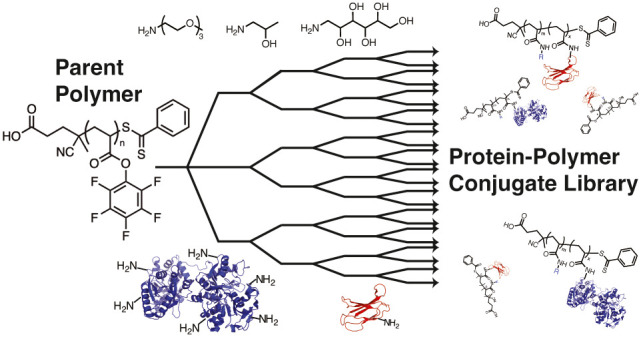

## Introduction

Protein–polymer conjugates are macromolecular bioconjugates that combine the biological specificity of proteins with the chemical tunability of synthetic polymers to address myriad challenges in chemistry, biotechnology, and medicine.^1^ For example, proteins are attractive for catalysis (i.e., enzymes), in consumer products, as vaccine components, as cell-targeting ligands for drug delivery, or as therapeutics themselves. However, many proteins lack the structural stability required for storage and transport.^2^ In addition, when used in therapeutic contexts, they are often cleared rapidly from the body or elicit an immune response that can limit their clinical use. Conjugation of synthetic polymers to proteins has been shown to improve protein stability under a range of conditions as well as prolong circulation half-lives and evade an immune response.^1–6^

Protein–polymer conjugates are generally synthesized either by polymerizing water-soluble monomers directly from a protein-based initiator (i.e., ‘grafting-from’) or coupling a presynthesized polymer bearing a reactive chain end to a protein (i.e., ‘grafting-to’).^1,5,7–9^ While these methods are useful for preparing well-defined protein–polymer conjugates, they require that new polymer structures be synthesized each time changes to the bioconjugate are desired. This may lead to variation in the polymer molecular weights and dispersities. Furthermore, grafting-from requires that proteins-of-interest be modified with polymerization initiators prior to polymerization, which makes it more difficult to assemble the bioconjugates in a modular or combinatorial way. Combinatorial synthetic approaches that permit rapid preparation of diverse biomaterial scaffolds can accelerate the identification of materials with improved properties.^10–12^ While libraries of protein–polymer conjugates have been prepared and characterized,^13–16^ to the best of our knowledge, few reports have described truly combinatorial approaches for synthesizing protein–polymer conjugates.

In the work described here, we sought to develop a combinatorial approach for synthesizing protein–polymer conjugates by sequential postpolymerization modification of side-chain reactive homopolymers. Postpolymerization modification of reactive homopolymers has several practical advantages for preparing functional materials such as protein–polymer conjugates.^11,17,18^ First, it obviates the need to synthesize a new polymer scaffold for each new functionality desired in the material. Rather, a parent polymer material with a defined molecular weight and dispersity can be modified with a variety of different functional groups to explore the influence of polymer structure on (bio)­material function. In addition, functionality that is difficult to incorporate into polymers using traditional polymerization methods can be added postpolymerization. Finally, in the context of protein–polymer conjugates, the number of protein-reactive groups along the backbone can easily be varied to investigate the influence of polymer reactivity on conjugation efficiencies and the resulting bioconjugate structures. Postpolymerization modification of reactive homopolymers has been used to prepare a broad range of multifunctional materials,^17–21^ including for the synthesis of libraries of polymers useful for nucleic acid delivery,^22,23^ peptide–polymer conjugates,^24^ thermally responsive materials,^25^ and functional polyacrylates.^26^

We recently reported the preparation of protein–polymer conjugates using this postpolymerization modification approach to materials synthesis.^27^ In this initial work, the azlactone-functionalized homopolymer poly­(2-vinyl-4,4-dimethylazlactone) (PVDMA) was partially modified with the hydrophilic alcohol triethylene glycol monomethyl ether (mTEG) to generate water-soluble, side-chain reactive copolymers.^27^ These polymers were conjugated to the iron-transport protein holo-transferrin (hTF) and the resulting structures were internalized by cells via receptor-mediated endocytosis.^27^ Other polymers bearing reactive side chains have also been used for protein conjugation,^28–36^ however, these materials were generally synthesized by copolymerization of a protein-reactive monomer with an inert, hydrophilic comonomer and not by postpolymerization modification of a single parent polymer.

Here, we describe the combinatorial synthesis of a small library of protein–polymer conjugates by sequential postpolymerization modification of the reactive homopolymer poly­(pentafluorophenyl acrylate) (PPFPA) (Scheme 1). PPFPA reacts rapidly with amine-functionalized nucleophiles and has been used extensively for the synthesis of functional materials.^18,37,38^ Because of their reactivity with lysine residues, activated pentafluorophenyl esters have also been used for conjugation to proteins on both the polymer side-chain^28^ and chain-terminus.^39^ While polymers bearing other activated esters such as *N*-hydroxysuccinimidyl (NHS) esters and azlactones have been used more extensively for conjugation to proteins,^1,5,8,27,29,40^ we elected to use PFP esters for this work for several reasons. First, NHS esters are known to be vulnerable to side reactions during functionalization, such as ring opening of the succinimide.^18^ PFP esters are also more resistant to hydrolysis^39,41^ and, in related work, have led to higher protein conjugation efficiencies than analogous NHS esters.^39^ Finally, using an activated ester with a fluorinated leaving group allows for straightforward characterization of reaction progress by ^19^F NMR spectroscopy.

**Scheme 1:**
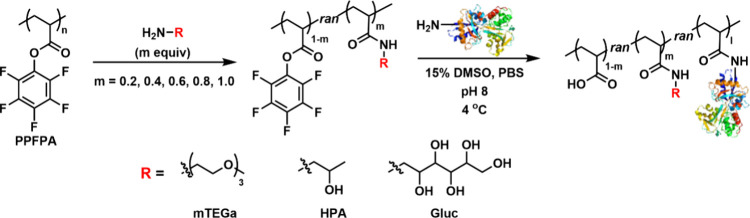
Combinatorial Synthesis of Protein–Polymer Conjugates by Postpolymerization Modification of PPFPA

In this work, we synthesized amphiphilic, protein-reactive copolymers by postpolymerization modification of PPFPA with three different hydrophilic amines at a range of grafting densities (Scheme 1). Functionalized polymers from the library that exhibited clarity in aqueous solution were conjugated to two different proteins of interest in the context of targeted drug delivery. The influence of polymer and protein structure on protein conjugation efficiencies and resulting bioconjugate structure are discussed. This work demonstrates a modular synthetic approach for preparing diversely functionalized protein–polymer conjugates that can be tailored to a broad range of different applications.

## Results and Discussion

### Polymer Synthesis and Characterization

The parent homopolymer PPFPA was synthesized from the vinyl monomer using RAFT polymerization with good control over the molecular weight and dispersity (Table 1, row 1). This homopolymer was used as a template for the synthesis of a library of 15 different random copolymers. We selected the hydrophilic amines methoxy-triethylene glycol amine (mTEGa), 1-amino-2-propanol (HPA), and glucamine (gluc) for postpolymerization modification of PPFPA. Polymers with side chains analogous to mTEGa and HPA have been proposed as biocompatible alternatives to the more commonly used linear poly­(ethylene glycol) (PEG) and have been used for the synthesis of other protein–polymer conjugates.^42,43^ Glucamine was also investigated in this context because it is hydrophilic and has been shown to resist the nonspecific adhesion of proteins, cells, and bacteria when used for the modification surfaces.^42,43^ Other glycopolymers have been used for protein–polymer conjugation;^41^ however, to the best of our knowledge, linear polyols like glucamine have not been used.

**Table 1:** Reactive Copolymers Synthesized for Protein Conjugation

polymer abbrev.	side chain^a^	target PFPA:R^b^	actual PFPA:R^c^	*M*_n,Theo_ (kg/mol)^d^	*M*_n,GPC_ (kg/mol)^e^	Đ^e^	aqueous soluble^g^
PPFPA				20.5	17.3	1.28	no
P(PFPA)_80_-mTEGa_20_	mTEGa	80:20	77:23	20.1	22.2	1.13	no
P(PFPA)_60_-mTEGa_40_	mTEGa	60:40	61:39	19.8	17.8	1.21	no
P(PFPA)_40_-mTEGa_60_	mTEGa	40:60	42:58	19.4	17.2	1.23	yes
P(PFPA)_20_-mTEGa_80_	mTEGa	20:80	22:78	19.1	21.8	1.11	yes
P(PFPA)_0_-mTEGa_100_	mTEGa	0:100	0:100	18.8	20.4	1.12	yes
P(PFPA)_80_-HPA_20_	HPA	80:20	80:20	18.7	17.9	1.28	no
P(PFPA)_60_-HPA_40_	HPA	60:40	63:37	17.1	16.4	1.31	no
P(PFPA)_40_-HPA_60_	HPA	40:60	43:57	15.3	17.1	1.25	yes
P(PFPA)_20_-HPA_80_	HPA	20:80	25:75	13.6	13.7	1.21	yes
P(PFPA)_0_-HPA_100_	HPA	0:100	0:100	11.3	n.d.^f^	n.d.	yes
P(PFPA)_80_-Gluc_20_	Gluc	80:20	76:24	20.5	n.d.	n.d.	yes
P(PFPA)_60_-Gluc_40_	Gluc	60:40	58:42	20.4	n.d.	n.d.	yes
P(PFPA)_40_-Gluc_60_	Gluc	40:60	42:58	20.4	n.d.	n.d.	yes
P(PFPA)_20_-Gluc_80_	Gluc	20:80	22:78	20.3	n.d.	n.d.	yes
P(PFPA)_0_-Gluc_100_	Gluc	0:100	0:100	20.3	n.d.	n.d.	yes

a Side chain structures and abbreviations are shown in Scheme 1.

b Target ratio was dictated by the mol % of amine relative to PFPA repeat unit added to the reaction.

c Actual ratio of amine incorporated into the polymer was determined by ^19^F NMR spectroscopy.

d Theoretical molecular weights calculated as described in the Supporting Information.

e Determined by GPC using THF as a solvent.

f Data not determined (n.d.) due to lack of solubility in available GPC solvents.

g These polymers produced clear solutions when dissolved in aqueous media and were used for protein conjugation.

Amphiphilic copolymers were synthesized by mixing PPFPA with substoichiometric equivalents of the amine relative to the activated ester (Table 1, rows 2–5, 7–10, and 12–16). We targeted copolymers containing side chain ratios of hydrophilic group:activated esters of 20:80, 40:60, 60:40, 80:20, and 100:0 to explore a wide range of reactive group densities. These polymers will be referred to by the targeted PFPA:hydrophilic group ratio. For example, PPFPA treated with 0.6 equiv of mTEGa relative to the PFPA unit is referred to as P­(PFPA)_40_-mTEGa_60_. The actual degree of functionalization was determined using ^19^F NMR spectroscopy (Figure 1). Prior to the addition of the amine, the ^19^F NMR spectrum of PPFPA exhibited only three broad signals for the PFPA repeat units (Figure 1, bottom spectrum). Upon addition of increasing equivalents of amine, the broad polymer peaks decrease in intensity while sharp peaks corresponding to the pentafluorophenol leaving group appear (Figure 1, 60%, 80%, and 100% mTEG functionalization). The relative integrations of the polymer and monomer peaks were used to determine the actual degree of functionalization. In general, the actual degree of functionalization correlated well with the targeted PFPA:hydrophilic group ratio (Table 1), demonstrating that copolymers with controlled compositions can be synthesized using this postpolymerization modification route.

**Figure 1:**
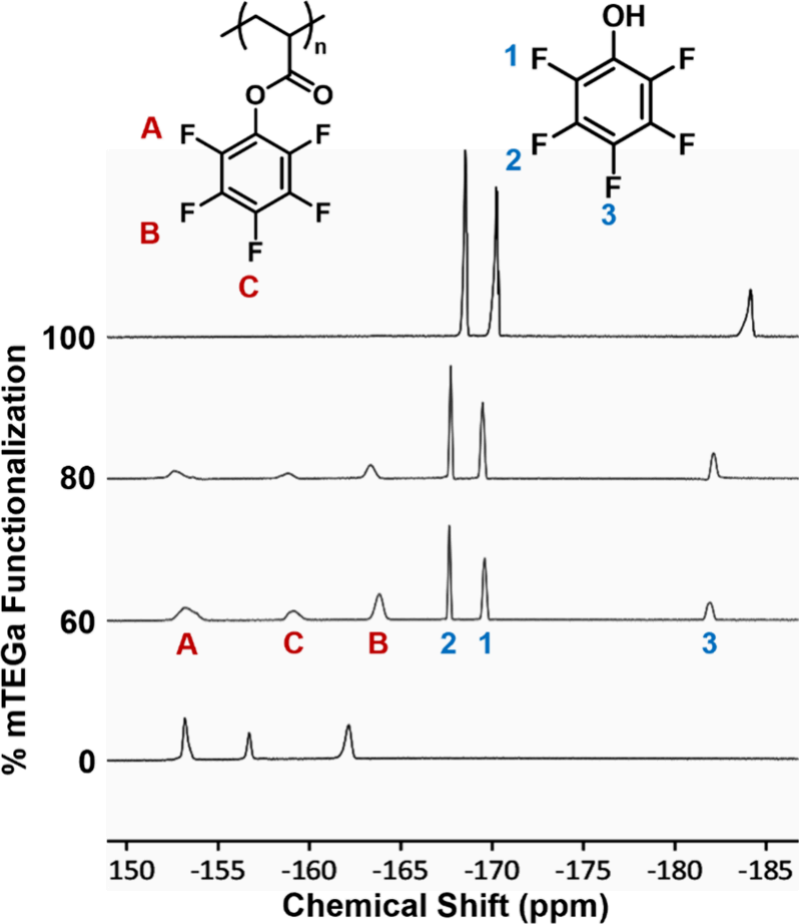
^19^F NMR spectra of PPFPA before and after functionalization with 0.6, 0.8, and 1.2 equivs of mTEGa.

Table 1 also shows the theoretical molecular weights, *M*_n,theo_, and polystyrene equivalent molecular weights determined from GPC, *M*_n,GPC_. *M*_n,theo_ for the PPFPA homopolymer was determined using monomer conversion, monomer:chain transfer agent ratio, and molecular weight of the monomer and end groups. *M*_n,theo_ for all functionalized polymers was determined using *M*_n,theo_ for PPFPA and adjusted to account for percent modification of the activated esters with each hydrophilic amine. Actual percent modifications determined using ^19^F NMR spectroscopy were used for these calculations. The *M*_n,GPC_ determined for the PPFPA and both mTEGa- and HPA-functionalized copolymers were similar to the theoretical molecular weights. Furthermore, the molecular weights and dispersities of the copolymers did not change substantially (Table 1 and Supporting Information), suggesting that the polymer chain length was not altered during the functionalization reactions. We did note the appearance of a high molecular weight shoulder in the GPC traces the for mTEGa-functionalized polymers (see Supporting Information). This shoulder may be a result of polymer aggregation or cross-linking either along the backbone or at the chain termini. Notably, we did not observe analogous high molecular weight shoulders for PPFPA-HPA polymers, which might be expected given that HPA has a pendant alcohol that could serve to cross-link the polymer chains. Current work is focused on understanding the structures and conformation of PPFPA-mTEG polymers in solution that might give rise to this shoulder. Unfortunately, P­(PFPA)_0_-HPA_100_ and all glucamine-modified polymers were not soluble in the solvents used for GPC and, thus, molecular weight determination was not possible. However, given that the *M*_n,GPC_ determined for all other copolymers did not vary significantly from the calculated *M*_n,theo_ (Table 1), we suspect that functionalization with glucamine would show similar molecular weight trends.

Polymers were next prepared for protein conjugation by first dissolving in DMSO near the polymer saturation limit prior to dissolving in PBS; maximizing the polymer concentration increases conjugation by increasing the probability of interactions with protein. Similar to our previous work,^27^ we used 15% DMSO/PBS (v/v) for solubilizing the polymers for subsequent conjugation to avoid denaturing protein with excess DMSO. Polymers that formed visibly clear solutions at concentrations in the range of ∼1 mg/mL to ∼150 mg/mL were used for protein conjugation experiments (Table 1). As shown in Table 1, mTEGa- and HPA-functionalized polymers yielded visibly clear solutions at modifications of 60% and above while all glucamine-functionalized polymers were soluble. It is important to note that visibly clear solutions likely contain higher order structures due to the high polymer concentration. We attempted characterization of these polymer structures using dynamic light scattering (DLS), however, the sizes of the structures were variable and inconsistent (data not shown). We attribute this variability to the high polymer concentrations used for conjugation. The maximum concentration that produced a clear solution depended on both the degree of functionalization and the side chain identity. In general, HPA-functionalized polymers were the least soluble and glucamine-functionalized polymers were the most soluble.

### Conjugation of Reactive Copolymers to Proteins

We next investigated the conjugation of our polymer library to two different proteins of interest in the context of targeted drug delivery. Building on our previous work,^27^ we selected holo-transferrin (hTF), which is an 80 kDa glycoprotein containing 58 lysine residues in addition to the N-terminus. It is the native protein ligand for the transferrin receptor, and we showed previously that hTF-polymer conjugates can be internalized into cells through receptor-mediated endocytosis.^27^ An 11.2 kDa protein derived from the tenth domain of fibronectin type III (termed Fn3) whose native sequence binds integrin receptors was selected as a smaller comparator. This engineered protein is produced in our laboratory informed by a previously reported Fn3 sequence engineered to bind αvβ3 integrin receptors with high affinity and specificity^44^ and has three solvent exposed lysine residues in addition to the N-terminus. We investigated the conjugation efficiency of Fn3 and hTF to all copolymers from our library (Table 1). For each polymer and protein combination, the influence of polymer:protein mol ratio, protein identity, side chain identity, and percent polymer functionalization on protein–polymer conjugation efficiencies was studied.

### Influence of Polymer:Protein Ratio on Conjugation Efficiency

The influence of polymer:protein mol ratio on conjugation efficiencies was first explored. Polymers that were 80% modified with the hydrophilic side chain (i.e., P­(PFPA)_20_-mTEGa_80_, P­(PFPA)_20_-HPA_80_, and P­(PFPA)_20_-Gluc_80_) were all soluble in the DMSO/PBS (15% v/v) at concentrations that yielded 1:1, 10:1, and 100:1 mol ratios of polymer:protein. The maximum mol ratio achievable for polymers modified with fewer hydrophilic side chains were lower and varied depending on the side chain; thus, the discussion here is focused on the set of polymers modified with 80% hydrophilic groups. Based on the degree of polymerization for PPFPA determined from GPC and ^1^H NMR data (i.e., ∼72 repeat units), polymers functionalized with 80% hydrophilic side chains have approximately 14 reactive groups that remain available for protein conjugation. Thus, at a mol ratio of 1:1 polymer:protein, there would be ∼14 reactive sites on the functionalized polymers available for reaction with amines on the proteins. Therefore, the ratio of reactive primary amines on the proteins to reactive activated ester groups on the functionalized polymers for 1 mol Fn3:1 mol PPFPA is 4 amines:14 esters and for 1 mol hTF: 1 mol PPFPA is 59 amines:14 esters.

Figure 2A shows representative SDS-PAGE gels stained with SimplyBlue for the conjugation of P­(PFPA)_20_-mTEGa_80_ to both Fn3 (top) and hTF (bottom). Each lane (protein only and conjugate) was loaded with the same amount of total protein (1 μg) such that the amount of free, unconjugated protein could be compared across samples. Similarly, polymer only samples were loaded at the same concentrations of polymer as in conjugate samples (lanes 2, 4, and 6) to compare the electrophoretic mobility of polymer only to the conjugates. The polymer samples only stained at the highest concentration used for protein conjugation (lane 6). Little conjugation of either protein to P­(PFPA)_20_-mTEGa_80_ at 1:1 polymer:protein mol ratios was observed (Figure 2A, lane 3 in both gels) as demonstrated by the strong band for free, unconjugated protein and the lack of streaking at higher molecular weights. However, upon increasing the polymer:protein ratio to 10:1, both hTF and Fn3 proteins exhibited significantly more conjugation (Figure 2A, lane 5 in both gels); conjugation is evidenced by the reduction in the intensity of the free protein bands as well as the appearance of high molecular weight streaks. At the highest protein:polymer ratio of 100:1, the unconjugated protein band disappeared entirely, suggesting 100% conjugation of the protein to polymer (Figure 2A, lane 7 in both gels). Finally, the last lane shows that if the PPFPA is fully functionalized with mTEGa (P­(PFPA)_0_-mTEGa_100_), no conjugation occurs, as supported by no reduction in the free protein bands and no higher molecular weight staining (Figure 2A, lanes 9 in both gels). These observations suggest the formation of covalent bonds between polymer and protein via reactions with the activated ester side chains. We observed similar trends for conjugation of Fn3 and hTF to P­(PFPA)_20_-HPA_80_ and P­(PFPA)_20_-Gluc_80_ (Figure S1).

**Figure 2:**
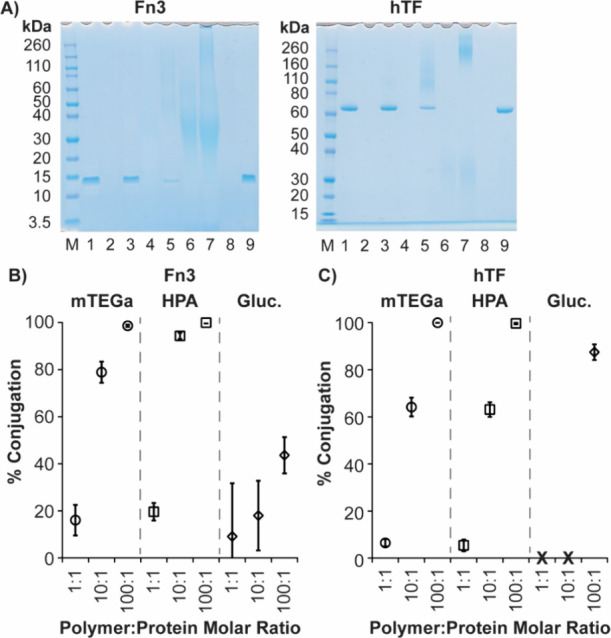
(A) Representative SDS PAGE gels for the conjugation of P­(PFPA)_20_-mTEGa_80_ to Fn3 (left) and hTF (right). Lane 1: protein only; Lane 2: polymer only (concentration used for 1:1 conjugation reaction); Lane 3: conjugation reaction (1:1 polymer:protein mol ratio); Lane 4: polymer only (concentration used for 10:1 polymer:protein mol ratio); Lane 5: conjugation reaction (10:1 polymer:protein mol ratio); Lane 6: polymer only (concentration used for 100:1 conjugation reaction); Lane 7: conjugation reaction (100:1 polymer:protein mol ratio); Lane 8: P­(PFPA)_0_-mTEGa_100_ only; Lane 9: conjugation reaction with P­(PFPA)_0_-mTEGa_100_. (B, C) Quantified protein conjugation efficiencies to (B) Fn3 and (C) hTF for P­(PFPA)_20_-mTEGa_80_, P­(PFPA)_20_-HPA_80_, and P­(PFPA)_20_-Gluc_80_ at various polymer:protein ratios. Data are represented as average conjugation efficiencies ± the standard deviation. Statistical analyses with *p*-values for these data are shown in Table S1.

We next quantified the conjugation efficiency of these reactions; conjugation efficiency refers to the percent of protein that was conjugated to polymer. We determined the conjugation efficiency by measuring the intensity of the unconjugated protein band in each PPC lane (Figure 2A and Figure S1, lanes 3, 5, and 7) and comparing that value with the intensity of the band in the protein only lane (Figure 2A and Figure S1, lane 1). Because the same amount of protein was loaded in each lane (i.e., 1 μg), the ratio of the intensity of the free protein band in each conjugation lane to the intensity of the protein band in protein only lane reflects the percentage of protein conjugated to polymer.

Figure 2B and Figure 2C show quantified conjugation efficiencies of Fn3 (Figure 2B) and hTF (Figure 2C) to all three polymers P­(PFPA)_20_-mTEGa_80_, P­(PFPA)_20_-HPA_80_, and P­(PFPA)_20_-Gluc_80_ as a function of polymer:protein ratio. Both Fn3 and hTF fully conjugated (i.e., 100% conjugation) to P­(PFPA)_20_-mTEGa_80_ and P­(PFPA)_20_-HPA_80_ at the highest polymer:protein mol ratio. In contrast, neither protein reached full conjugation upon reaction with P­(PFPA)_20_-Gluc_80_, even at the highest polymer:protein mol ratios. We will discuss the influence of side-chain identity on conjugation efficiencies in more detail in subsequent sections, however, these results demonstrate that complete consumption of protein is possible at high polymer:protein mol ratios for mTEGa- and HPA-functionalized polymers.

Increased polymer to protein ratio also impacts conjugate species formed. Closer inspection of the SDS-PAGE gels for these reactions suggests that different molecular populations form depending on the concentration used (Figure 2A for mTEGa and S1A for HPA). For example, reactions of P­(PFPA)_20_-mTEGa_80_ with hTF at a mol ratio of 10:1 yielded a conjugate streak centered around ∼100 kDa (Figure 2A, bottom gel, lane 5). Using an average molecular weight of ∼100 kDa for the conjugate, an apparent molecular weight of 66 kDa for hTF (based on the free protein band), and a molecular weight of ∼22 kDa for P­(PFPA)_20_-mTEGa_80_ based on GPC data, this conjugate contains an estimated average of 1.5 polymer chains per protein. However, upon increasing the polymer:protein mol ratio to 100:1 for the conjugation reaction, the PPC formed runs at ∼260 kDa (Figure 2A, bottom gel, lane 7), which is consistent with much larger structures (i.e., several polymers per protein) or cross-linked aggregates. Although the bands are more diffuse for the Fn3 conjugates, a similar trend was observed (Figure 2A, top gel). These results suggest that it is possible to tailor the bioconjugate structure depending upon the mol ratio of polymer:protein used. We note in this context that these reactive copolymers could also be used to fabricate protein-laden hydrogels. It is important to acknowledge that, although SDS was used to reduce noncovalent interactions between macromolecules, some of the species observed may be noncovalently associated aggregates. This would be most probable for conjugates with lower percent functionalization (more hydrophobic) and higher polymer:protein ratios (closer to saturation limit of the polymer). Future work will focus on isolating these conjugates and characterizing these structures.

### Influence of Side-Chain Grafting Density on Protein Conjugation Efficiency

We next sought to elucidate the influence of side-chain grafting density (or percent polymer modification) on protein conjugation. Here, conjugation efficiencies at polymer:protein mol ratios of 10:1 were used because these reactions showed greater variability in conjugation efficiencies and thus conclusions about the influence of polymer structure on conjugation could be made. Figure 3A shows representative SDS-PAGE gels for mTEGa-functionalized polymers at 60% (lanes 2), 80% (lanes 3), and 100% (lanes 4) polymer modification conjugated to Fn3 (left gel) and hTF (right gel). Figure 3B–D shows conjugation efficiencies for mTEGa-, HPA-, and glucamine-functionalized polymers, respectively, at 60, 80, and 100% polymer modifications. Both mTEGa- and glucamine-functionalized polymers exhibited reduced conjugation efficiencies as the degree of functionalization increased. These results are consistent with a reduction in the overall concentration of activated esters available for coupling and increased steric hindrance, both of which should reduce reaction efficiencies. In addition, polymers completely modified with the hydrophilic side chains yielded no detectable conjugation, as noted by the symbol ‘X’ in Figure 3B–D, due to a complete lack of reactive groups on the polymer. These results suggest that the conjugates formed in these reactions are a result of covalent coupling and not physical association.

**Figure 3:**
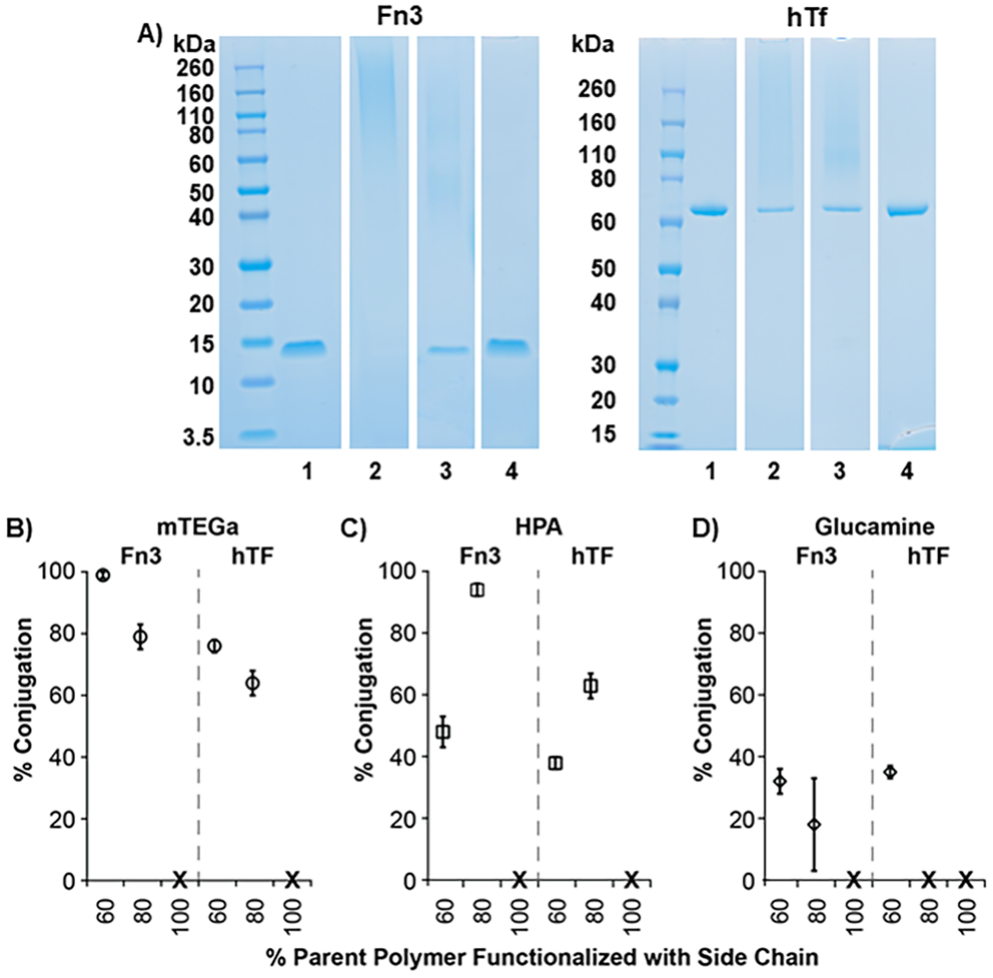
(A) Representative SDS PAGE gels showing conjugation of mTEGa-functionalized polymers at 60% (lane 2), 80% (lane 3), and 100% (lane 4) modification to Fn3 (left) and hTF (right). (B–D) Quantified protein conjugation efficiencies to Fn3 and hTF as a function of side chain density for (B) mTEGa-, (C) HPA-, and (D) glucamine-functionalized polymers. Data are represented as average conjugation efficiencies ± the standard deviation. Statistical analyses with *p*-values for these data are shown in Table S2.

Somewhat unexpectedly, HPA-functionalized copolymers exhibited more efficient conjugation at 80% functionalization relative to 60% functionalization (Figure 3C) for both Fn3 and hTF conjugation reactions. We speculate that this trend is due to reduced solubility of P­(PFPA)_40_-HPA_60_ in solution relative to P­(PFPA)_20_-HPA_80_ at polymer:protein mol ratios of 10:1. It is likely that aggregation or assembly of the polymers in solution reduced the overall concentration of available reactive groups, leading to lower-than-expected conjugation efficiencies. Although less pronounced, closer inspection of the SDS-PAGE gels for mTEG-functionalized copolymers showed streaking at higher molecular weights for P­(PFPA)_40_-mTEGa_60_-Fn3 conjugates relative to P­(PFPA)_20_-mTEGa_80_-Fn3 conjugates (Figure 3A). These results are similar to our experiments investigating the influence of polymer:protein mol ratio in which larger mol ratios favored higher molecular weight species. Here, increasing the reactive group density on the polymer, which effectively increases the concentration of reactive groups in solution, at the same polymer:protein mol ratio also favors the formation of higher molecular weight conjugate species. Given the excess of activated esters relative to protein amines for these reactions, it is likely that these higher molecular weight species represent two or more polymer chains conjugated to the Fn3. These results again suggest that it might be possible to tailor the structure of the conjugates formed by varying the number of reactive groups on the polymer.

### Influence of Side-Chain Identity on Protein Conjugation Efficiency

Further analysis of the data shown in Figure 3 reveals the ways in which the hydrophilic side chain chemistry influences the efficiency of protein conjugation. As discussed above, P­(PFPA)_40_-HPA_60_ exhibits reduced solubility relative to other polymers investigated here, so it is difficult to draw conclusions about the effect of side-chain identity at this degree of modification. However, comparison of all three side chains at 80% modification reveals that P­(PFPA)_20_-HPA_80_ yields slightly statistically higher conjugation efficiencies to Fn3 (94%) than does either P­(PFPA)_20_-mTEGa_80_ (79%) or P­(PFPA)_20_-Gluc_80_ (18%). Because HPA is substantially smaller than both mTEG and glucamine, it is unsurprising that conjugation efficiencies are higher for these copolymers. The smaller side chains likely offer greater accessibility to the activated ester.

Despite similar molecular weights between mTEG and glucamine, glucamine-modified polymers exhibited substantially reduced conjugation efficiencies relative to mTEG-functionalized polymers (Figure 3). We suspect that the branched hydroxyls on glucamine create a larger hydration sphere than do the ethers in mTEG, thus shielding neighboring activated esters from reaction with proteins. However, because glucamine is so hydrophilic, it is possible to reduce the degree of functionalization to achieve improved conjugation. All polymers modified with glucamine were at least somewhat soluble in water, even at percent modifications as low as 20% (Table 1). Figure 4 shows conjugation efficiencies of Fn3 to glucamine-functionalized polymers as a function of percent modification and polymer:protein ratio. Comparing conjugation efficiencies of these three polymers at the same polymer:protein ratio (i.e., 10:1), increasing the density of glucamine side chains on the polymer yielded reduced conjugation efficiencies.

**Figure 4:**
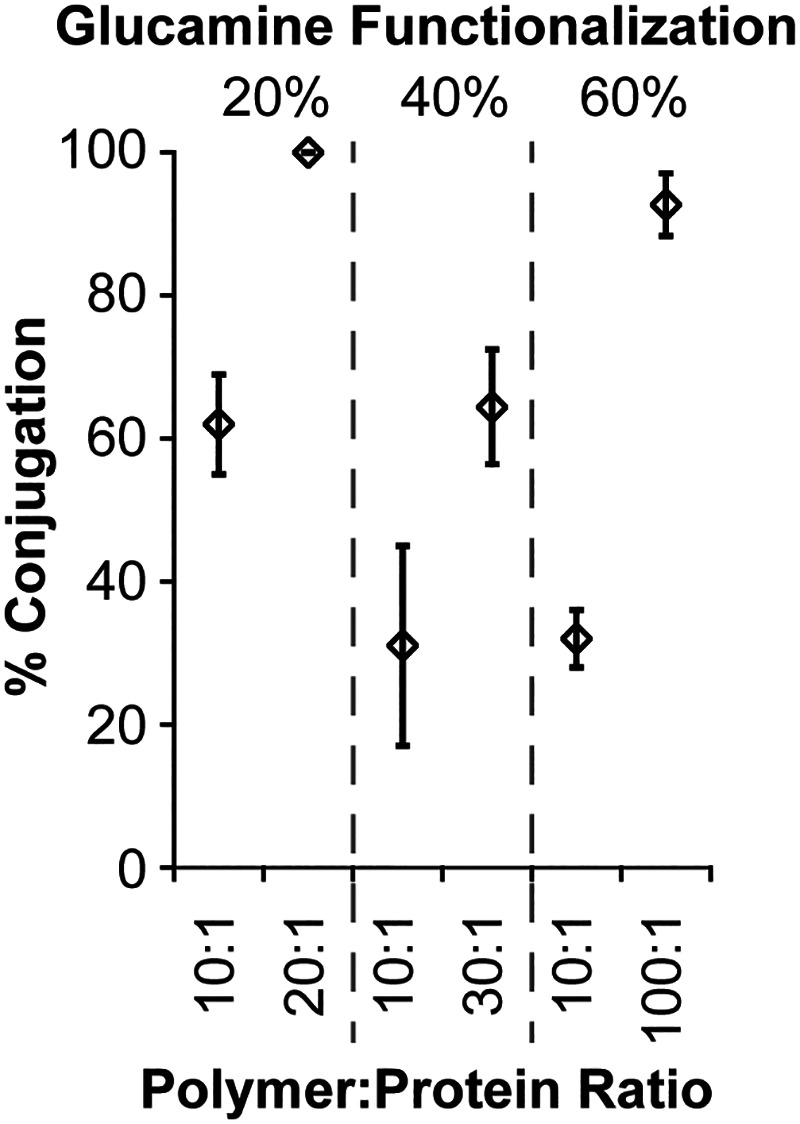
Quantified protein conjugation efficiencies of glucamine-functionalized polymers to Fn3 at 20, 40, and 60% functionalization at various polymer:protein ratios. Statistical analyses with *p*-values for these data are shown in Table S3.

These observations with P­(PFPA)-Gluc trends are consistent with the data described above. Increasing the polymer:protein ratio also improves conjugation efficiencies, as expected. To achieve 100% conjugation effciency, polymers were dissolved close to their saturation limits; the highest concentrations achievable for these polymers depended on the degree of functionalization. P­(PFPA)_80_-gluc_20_ showed nearly 100% conjugation of Fn3 at a polymer:protein mol ratio of 20:1. Similarly, P­(PFPA)_40_-gluc_60_ demonstrated nearly 100% conjugation at 100:1 polymer:protein mol ratio. P­(PFPA)_60_-gluc_40_ showed significantly reduced conjugation at the highest achievable polymer:protein mol ratio of 30:1. While the reasons for this trend are not entirely clear, it is possible that the polymer either was not entirely soluble despite visually clear solutions or that this polymer:protein mol ratio is not high enough to achieve full conjugation at this grafting density. Taken together, these data demonstrate that glucamine functionalization offers access to a wider range of polymer structures that can be conjugated to proteins in aqueous media and that high yields of conjugate can be achieved depending on the percent functionalization and polymer:protein mol ratio.

### Influence of Protein Identity on Conjugation Efficiency

Finally, the data shown in Figure 3 reveals that, in general, Fn3 couples more efficiently than does hTF to polymers with similar degrees of modification. This improvement is particularly notable given that the concentration of amines in the hTF conjugation reactions was nearly eight times higher than the concentration of amines in the Fn3 conjugation reactions. These results can be explained by the fact that Fn3 is significantly smaller than hTF and, thus, reactions between protein and polymer are more efficient. Further, conjugation efficiencies of P­(PFPA)_20_-HPA_80_ and P­(PFPA)_20_-mTEGa_80_ to the larger hTF protein were nearly identical at 63% and 64%, respectively, suggesting that protein size had a more significant effect on conjugation efficiencies than side-chain identity for these reactions.

## Conclusions

In summary, we have demonstrated a combinatorial approach to efficiently and modularly assemble protein–polymer conjugates. The synthetic strategy used here is simple and permits a diversity of structures to be prepared from a single starting polymer. Our results reveal a number of structural factors to consider when optimizing these reactions for both conjugation efficiency and conjugate structure. While increasing the ratio of polymer:protein in the conjugation reaction certainly improves the amount of protein conjugated to polymer, it favors the formation of higher molecular weight species that likely have multiple polymer chains linked to the protein or even cross-linked structures. Depending on the intended application, this may or may not be desirable. Similarly, increasing the number of reactive groups on the copolymer chain improves conjugation efficiencies when the polymer is sufficiently soluble in aqueous solution but also favors higher molecular weight species. Using shorter, less sterically bulky side chains (e.g., HPA) also facilitates improved conjugation. Although conjugation to glucamine-functionalized polymers is less efficient with increased functionalization, glucamine offers an exciting alternative to more commonly used hydrophilic copolymers. Because it is so hydrophilic, glucamine can solubilize hydrophobic species and may be useful for the delivery of hydrophobic drugs. While our work focused on preparing protein–polymer conjugates for targeted drug delivery applications, our synthetic approach is useful for the preparation of diverse bioconjugate structures of interest in a broad range of applications.

## Experimental Section

### Materials

All reagents were used as received unless otherwise noted. Pentafluorophenol, acryloyl chloride (>97%), triethylamine, 4-cyano-4-(phenylcarbonothioylthio)­pentanoic acid, 2,2′-azobis­(2-methylpropionitrile) (98%), (±) 1-amino-2-propanol, anhydrous 1,4-dioxane, THF, dichloromethane, hexanes, and ethyl acetate were purchased from Sigma-Aldrich. 2,2′-azobis­(2-methylpropionitrile) (98%) (AIBN) was recrystallized twice from methanol prior to use. mPEG3 amine (mTEGa) was purchased from BroadPharm. d-glucamine (>97%) was purchased from TCI America (Philadelphia, PA). All NMR solvents were purchased from Cambridge Isotope Laboratories. Column chromatography was performed with Sorbitech standard grade 60 Å silica gel. Holo-transferrin (hTF, Cat.: 616397) protein was purchased from CalBiochem. HisPur cobalt resin, NuPAGE 4–12% Bis-Tris gels, MES buffer, MOPS buffer, and LDS buffer were purchased from ThermoFisher Scientific. All solutions were made with ultrapure water from a ThermoScientific Barnstead Nanopure water filtration system unless noted otherwise.

### Instrumentation

^1^H (^19^F) NMR spectra were acquired on a Bruker 500 (470) MHz spectrometer at room temperature. Chemical shifts (δ) are reported in ppm relative to residual solvent peaks. Gel permeation chromatography (GPC) was performed on a Shimadzu Analytical UHPLC instrument equipped with a Shim-pack 803 column, operating in THF at 40 °C at a flow rate of 1 mL/min. Molecular weights and dispersities were measured against polystyrene calibration standards. Anhydrous THF and dichloromethane were obtained from Sigma-Aldrich and purified on an alumina column solvent purification system by LC Technology Solutions.

### Synthesis of Pentafluorophenyl Acrylate (PFPA)

PFPA was synthesized as previously described^45^ with minor modifications. Briefly, pentafluorophenol (20.0 g, 0.108 mol, 1 equiv) was weighed into an oven-dried 500 mL 3-neck round-bottom flask equipped with a stir bar and an oven-dried pressure-equalizing addition funnel. Anhydrous dichloromethane (200 mL) was added to the flask and the apparatus was placed under N_2_. Triethylamine (22.9 mL, 0.164 mol, 1.5 equiv) was added slowly to the flask, and the reaction solution was cooled to 0 °C in an ice bath. Acryloyl chloride (13.5 mL, 0.166 mol, 1.5 equiv) was added dropwise via the addition funnel over the course of an hour. The reaction was allowed to stir at room temperature for 24 h. The reaction was filtered to remove precipitate and the filtrate volume was reduced by half using rotary evaporation. The filtrate was washed with water (2 × 50 mL) and brine (2 × 50 mL) and dried with magnesium sulfate. The crude product was purified by flash column chromatography (20% DCM in hexanes) to yield a clear liquid (13.82 g, 53% yield). The pure product was stored at −20 °C.

^**1**^ **H NMR:** (300 MHz, CDCl_3_, 298 K; δ, ppm): 6.75 (dd, 1H), 6.39 (dd, 1H), 6.20 (dd, 1H)

^**19**^ **F NMR:** (282.231 MHz, CDCl3, 298 K; δ, ppm): −152.6 (d, 2F), −158.0 (t, 1F), −162.4 (t, 2F)

### Synthesis of Poly­(pentafluorophenyl acrylate) (PPFPA)

The chain transfer agent 4-cyano-4-(phenylcarbonothioylthio)­pentanoic acid (0.081 g, 0.291 mmol, 1 equiv) was weighed into an oven-dried round-bottom flask equipped with a stir bar. Pentafluorophenyl acrylate (6.92 g, 29.1 mmol, 100 equiv) was added to the flask, followed by anhydrous 1,4-dioxane (6 mL). Recrystallized AIBN (0.0048 g, 0.029 mmol, 0.1 equiv) dissolved in anhydrous 1,4-dioxane (0.3 mL) was added to the reaction. The flask was capped with a rubber septum and the reaction mixture was sparged with nitrogen for approximately 1 h. The reaction was heated to 70 °C for 13 h (83% conversion). The viscous reaction mixture was diluted in a minimal volume of THF (∼4 mL), and the polymer was precipitated twice into cold methanol to yield a light pink powder (4.872 g, 83.8% yield).

^**1**^ **H NMR:** (CDCl3, 298 K; δ, ppm): 3.10 (br s, 1H), 2.51 (br s, 0.5H), 2.14 (br m, 1.5H)

^**19**^ **F NMR:** (CDCl3, 298 K; δ, ppm): −153.72 (br s, 2F), −156.73 (br s, 1F), −162.17 (br s, 2F)

**GPC:** *M*_n_ = 17.3 kg/mol; Đ = 1.28

### Postpolymerization Modification of PPFPA with mTEGa

All PPFPA-mTEGa copolymers were synthesized according to the following general procedure. PPFPA (100 mg, 0.42 mmol with respect to the PFPA repeat unit, 1 equiv) was weighed into an oven-dried, 10 mL round bottomed flask equipped with a stir bar and dissolved in anhydrous THF (2 mL). The flask was capped with a septum and sparged with N_2_ for 10 min mTEGa (eq determined by target modification) was dissolved in anhydrous THF (2 mL) and added dropwise to the stirring PPFPA solution via a syringe. The reaction was stirred at 50 °C for various times depending on the target modification (see details for each polymer). Prior to precipitation, percent polymer modification was determined using ^19^F NMR spectroscopy with a delay time of 7 s and an acquisition time of 4 s. The reaction was stopped when the target degree of polymerization was reached as determined using the following equation: % func. = [integral­(PFP–OH)]/[integral­(PFP–OH) + integral­(polymer-PFP)] × 100. The polymer was then precipitated twice into a mixture of hexanes and diethyl ether.

#### P­(PFPA)_0.8_-mTEGa_0.2_

mTEGa (13.7 mg, 0.084 mmol, 0.2 eq with respect to the PFPA repeat unit). The reaction was stirred for 3 h. Functionalized polymer was precipitated twice into 5:1 diethyl ether:hexanes.

#### P­(PFPA)_0.6_-mTEGa_0.4_

mTEGa (27.4 mg, 0.168 mmol, 0.4 eq with respect to the PFPA repeat unit). The reaction was stirred for 3 h. Functionalized polymer was precipitated twice into 2:1 diethyl ether:hexanes.

*P­(PFPA)*_0.4_ *-mTEGa*_0.6_. mTEGa (41.1 mg, 0.252 mmol, 0.6 eq with respect to the PFPA repeat unit). The reaction was stirred for 5 h. Functionalized polymer was precipitated twice into 1:1 diethyl ether:hexanes.

#### P­(PFPA)_0.2_-mTEGa_0.8_

mTEGa (54.8 mg, 0.336 mmol, 0.8 eq with respect to the PFPA repeat unit). The reaction was stirred for 25 h. Functionalized polymer was precipitated twice into 4:1 diethyl ether:hexanes.

#### P­(PFPA)_0_-mTEGa_100_

mTEGa (82.1 mg, 0.504 mmol, 1.2 eq with respect to the PFPA repeat unit). The reaction was stirred for 3 h. Functionalized polymer was precipitated twice into 4:1 diethyl ether:hexanes.

### Postpolymerization Modification of PPFPA with HPA

All PPFPA-HPA copolymers were synthesized according to the following general procedure. PPFPA (100 mg, 0.42 mmol with respect to the PFPA repeat unit, 1 equiv) was weighed into an oven-dried, 4 mL vial equipped with a stir bar and dissolved in anhydrous THF (0.25 mL). The vial was capped with a septum and sparged with N_2_ for 10 min. HPA (eq determined by target modification) was dissolved in anhydrous THF (0.25 mL) and added slowly and dropwise to the stirring PPFPA solution via a syringe. The reaction was stirred at room temperature for various times depending on the target modification (see details for each polymer). Prior to precipitation, percent polymer modification was determined using ^19^F NMR spectroscopy with a delay time of 7 s and an acquisition time of 4 s. The reaction was stopped when the target degree of polymerization was reached as determined using the following equation: % func. = [integral­(PFP–OH)]/[integral­(PFP–OH) + integral­(polymer-PFP)] × 100. The polymer was then precipitated twice into solvents that varied based on the percent modification.

#### P­(PFPA)_0.8_-HPA_0.2_

HPA (6.3 mg, 0.084 mmol, 0.2 eq with respect to the PFPA repeat unit). The reaction was stirred overnight. Functionalized polymer was precipitated twice into hexanes.

#### P­(PFPA)_0.6_-HPA_0.4_

HPA (12.6 mg, 0.168 mmol, 0.4 eq with respect to the PFPA repeat unit). The reaction was stirred overnight. Functionalized polymer was precipitated twice into hexanes.

#### P­(PFPA)_0.4_-HPA_0.6_

HPA (19.0 mg, 0.252 mmol, 0.6 eq with respect to the PFPA repeat unit). The reaction was stirred overnight. Functionalized polymer was precipitated twice into hexanes.

#### P­(PFPA)_0.2_-HPA_0.8_

HPA (25.2 mg, 0.336 mmol, 0.8 eq with respect to the PFPA repeat unit). The reaction was stirred overnight. Functionalized polymer was precipitated twice into diethyl ether.

#### P­(PFPA)_0_-HPA_100_

HPA (63.1 mg, 0.840 mmol, 2 eq with respect to the PFPA repeat unit). The reaction was stirred overnight. Functionalized polymer was precipitated twice into diethyl ether.

### Postpolymerization Modification of PPFPA with Glucamine

All PPFPA-Gluc copolymers were synthesized according to the following general procedure. PPFPA (200 mg, 0.84 mmol with respect to the PFPA repeat unit, 1 equiv) was weighed into an oven-dried, 8 mL vial equipped with a stir bar and dissolved in anhydrous DMF (3 mL). The vial was capped with a septum and sparged with N_2_ for 10 min. Glucamine (equiv determined by target modification) was dissolved in anhydrous DMF (3 mL) and added slowly and dropwise to the stirring PPFPA solution via a syringe. The reaction was stirred at 50 °C for various times depending on the target modification (see details for each polymer). Prior to precipitation, percent polymer modification was determined using ^19^F NMR spectroscopy with a delay time of 7 s and an acquisition time of 4 s. The reaction was stopped when the target degree of polymerization was reached as determined using the following equation: % func. = [integral­(PFP–OH)]/[integral­(PFP–OH) + integral­(polymer-PFP)] × 100. The polymer was then precipitated twice into solvents that varied based on the percent modification.

#### P­(PFPA)_0.8_-Gluc_0.2_

Glucamine (30.4 mg, 0.168 mmol, 0.2 eq with respect to the PFPA repeat unit). The reaction was stirred for 2 h. Functionalized polymer was precipitated from DMF once into 1:1 petroleum ether:ethyl acetate and once into 1:2 petroleum ether:ethyl acetate.

#### P­(PFPA)_0.6_-Gluc_0.4_

Glucamine (60.9 mg, 0.336 mmol, 0.4 eq with respect to the PFPA repeat unit). The reaction was stirred for 2 h. Functionalized polymer was precipitated from DMF once into 1:1 petroleum ether:ethyl acetate and once into 1:5 petroleum ether:ethyl acetate.

#### P­(PFPA)_0.4_-Gluc_0.6_

Glucamine (91.4 mg, 0.504 mmol, 0.6 eq with respect to the PFPA repeat unit). The reaction was stirred for 3 h. Functionalized polymer was precipitated from DMF once into 1:5 petroleum ether:ethyl acetate and once into ethyl acetate.

#### P­(PFPA)_0.2_-Gluc_0.8_

Glucamine (182.0 mg, 1.01 mmol, 1.2 eq with respect to the PFPA repeat unit). The reaction was stirred for 3 h. Functionalized polymer was precipitated twice into ethyl acetate.

#### P­(PFPA)_0_-Gluc_100_

Glucamine (182.0 mg, 1.01 mmol, 1.2 eq with respect to the PFPA repeat unit). The reaction was stirred for 24 h. Functionalized polymer was precipitated twice into ethyl acetate.

### Expression and Purification of Fn3 Protein

An Fn3 variant with high specificity for αvβ3 integrin receptors was identified based on the high affinity consensus sequence determined by Richards et al. using phage display.^44^ The modified RGD sequence in the FG binding loop was PRGDWNEG. Fn3 protein was expressed and purified using protocols based on those we reported previously.^46,47^ Briefly, a gene coding for the high affinity protein was cloned into a pETh protein expression vector containing a C-terminus hexahistidine tag for metal affinity purification. The sequence was confirmed by Sanger sequencing. The plasmid was transformed into BL21­(DE) *E. coli*. Protein expression was induced by IPTG with shaking at 37 °C for 3 h, and bacteria were subsequently lysed by repeated freezing and thawing. Supernatant was recovered by centrifugation and then filtered using 0.45- and 0.22-μm syringe filters. Protein was purified using HisPur cobalt resin. Purified protein was buffer exchanged into PBS and concentrated using a centrifugal filtration device with 3 kDa molecular weight cutoff. Purity of Fn3 protein was assessed using SDS-PAGE.

### Synthesis of Protein–Polymer Conjugates

The following general procedure was used for all conjugation reactions. Polymer stock solutions were prepared in DMSO at concentrations of 37.7 mM, 3.77 mM, and 0.377 mM to be used for conjugation experiments conducted at 100:1, 10:1, and 1:1 mol ratios of polymer:protein, respectively. These concentrations were selected to yield a final DMSO concentration that did not exceed 15% (v/v) in the conjugation reaction solutions. For other polymer:protein ratios (e.g., 20:1 and 30:1), the concentration of the stock polymer solution was adjusted accordingly. The mass of polymer used for these solutions was calculated using the *M*_n_ determined from GPC. For polymers that could not be characterized using GPC due to solubility, the theoretical *M*_n_ was used. Protein in PBS was added to the conjugation reaction tubes to achieve a final concentration of 1 mg protein/1 mL total reaction solution. Sodium bicarbonate (1 M) was added to the conjugation solutions to a final concentration of 0.1 M to increase the nucleophilicity of primary amines. Polymer stock in DMSO was added to achieve the target molar ratio of polymer:protein. Protein control mixtures were assembled by replacing the polymer solution with an equivalent volume of DMSO, and polymer control mixtures were assembled by replacing the protein solution with an equivalent volume of PBS. The solutions were mixed well in 1.5 mL microcentrifuge tubes and rotated at 4 °C for 7 days. A 1-week time point was used to both maximize the conjugation due to the hydrophobic nature of PFP and hydrolyze unreacted PFP repeats. The reaction was mixed at 4 °C to ensure protein stability throughout the study.

Conjugate mixtures were not purified prior to SDS-PAGE as keeping the original components of the mixture was necessary to determine conjugation efficiency. Aliquots of the conjugation mixtures were combined with NuPAGE LDS Sample Buffer and PBS such that the concentration of protein was 0.1 mg/mL. Samples were heated for 10 min at 70 °C to denature proteins and then loaded onto a NuPAGE 4–12% Bis-Tris gel along with a Novex Sharp Protein reference ladder. All protein-containing lanes were loaded with 1 μg of protein. Polymer only lanes were loaded with the equivalent concentration of polymer that was used for conjugation experiments. Gel electrophoresis was performed in MOPS buffer for 50 min with a constant voltage of 200 V for hTf and MES buffer for 35 min with a constant voltage of 200 V for Fn3. The gel was stained with SimplyBlue SafeStain (30 min) and imaged using a Canon Pixma scanner. To determine conjugation efficiency, the intensities of Fn3 and transferrin bands (∼11 and 80 kDa, respectively) in the conjugate lanes were quantified using the FIJI gel analysis function, and all data were normalized to their respective protein-only control lanes.

### Statistics

All conjugate summary data are represented as means ± standard deviation (*n* = 3 conjugate reactions). Statistical differences between groups were determined using unpaired Student’s two-tailed *t* tests analyzed using GraphPad Prism. All *p*-values for data presented in figures are reported in the Supporting Information.

## Supplementary Material



## Notes

Representative SDS-PAGE gels for P­(PFPA)_20_-HPA_80_ and P­(PFPA)_20_-Gluc_80_ to Fn3 and hTf; *p*-values from *t* tests; representative ^1^H and ^19^F NMR spectra for homopolymer and functionalized polymers; representative GPC traces for homopolymer and functionalized polymers (PDF)
