# Supplementary material for: Combinatorial Synthesis of Protein–Polymer Conjugates by Postpolymerization Modification of Poly(pentafluorophenyl acrylate)s
Source: ACS Omega. 2026 Jan 8;11(3):3926–36. doi: 10.1021/acsomega.5c07215 (PMC12854635; doi:10.1021/acsomega.5c07215)
Supplement: Supplementary file 1 [file ao5c07215_si_001.pdf]

SUPPORTING INFORMATION

for

Combinatorial Synthesis of Protein-Polymer Conjugates by Post-Polymerization Modification of Poly(Pentafluorophenyl Acrylate)s.

Emily W. Kish,[1‡] Thatcher M. Lee,[1‡] Alexis M. Ziemba[2‡], Ama Boamah,[1] Bianca Figueroa,[1] Margherita Piccardi,[3,4] Carey E. Dougan,[1] Sarah J. Moore,[4] Maren E. Buck[1*]

[1] Department of Chemistry, Smith College, Northampton, MA.
[2] Neuroscience Program, Smith College, Northampton, MA.
[3] Department of Biology, University of Pisa, Pisa, Italy
[4] Picker Engineering Program, Smith College, Northampton, MA.

***Corresponding Author**
E-mail: mbuck@smith.edu

TABLE OF CONTENTS

Figure S1. SDS PAGE gels for HPA- and glucamine-functionalized polymers S2

Table S1. Statistical analyses for Figure 2 S3

Table S2. Statistical analyses for Figure 3 S4

Table S3. Statistical analyses for Figure 4 S4

Figures S2-S12: Representative NMR spectra S5

Figures S13-S22: Representative GPC traces S11

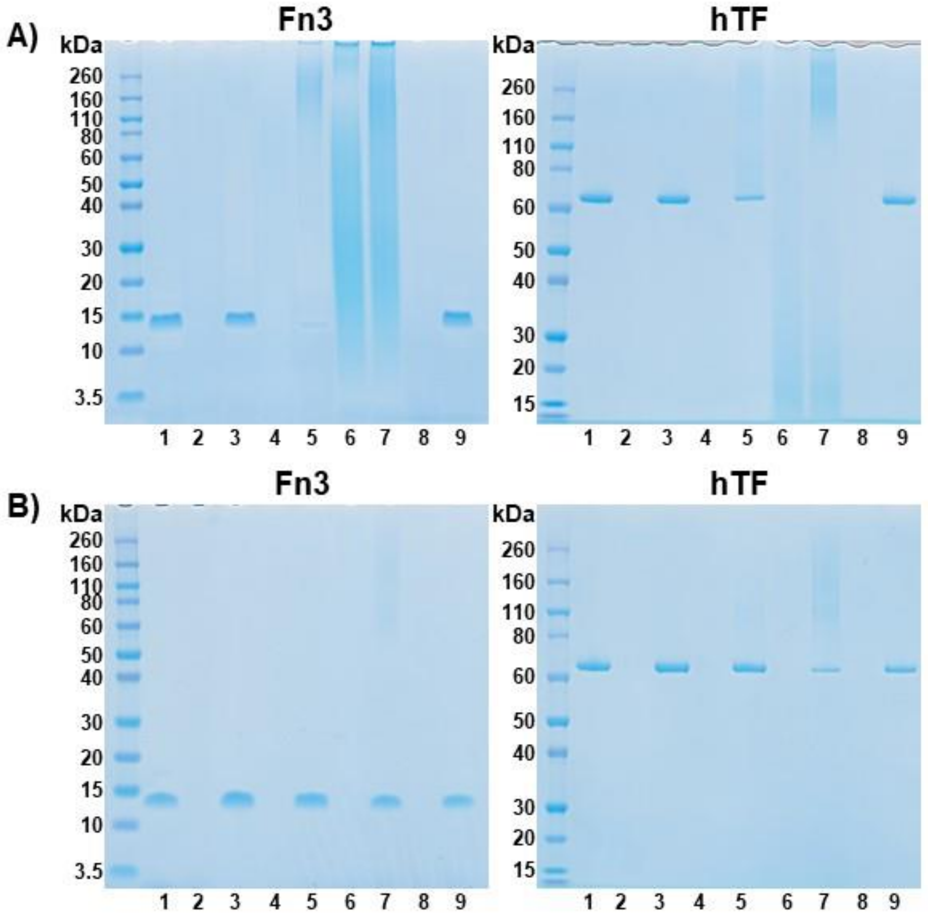

Figure S1. A) Representative SDS PAGE gels for the conjugation of P(PFPA)$_{20}$-HPA$_{80}$ to Fn3 (left) and hTF (right). B) Representative SDS PAGE gels for the conjugation of P(PFPA)$_{20}$-Gluc$_{80}$ to Fn3 (left) and hTF (right). Lane 1: protein only; Lane 2: polymer only (concentration used for 1:1 conjugation reaction); Lane 3: conjugation reaction (1:1 polymer:protein mol ratio); Lane 4: polymer only (concentration used for 10:1 polymer:protein mol ratio); Lane 5: conjugation reaction (10:1 polymer:protein mol ratio); Lane 6: polymer only (concentration used for 100:1 conjugation reaction); Lane 7: conjugation reaction (100:1 polymer:protein mol ratio); Lane 8: 100% functionalized polymers only; Lane 9: conjugation reaction with 100% functionalized polymers. Data are represented as average conjugation efficiencies ± the standard deviation. Statistical analyses with p-values for these data are shown in Table S1.

Table S1. P-values from t-tests of samples shown in Figure 2.

Sample 1			Sample 2			p-value[c]	Interpretation[d]
Protein	Side Chain[a]	Pol:Prot[b]	Protein	Side Chain[a]	Pol:Prot[b]		
Fn3	mTEGa	1:1	Fn3	mTEGa	10:1	0.0002	***
Fn3	mTEGa	1:1	Fn3	mTEGa	100:1	< 0.0001	***
Fn3	mTEGa	1:10	Fn3	mTEGa	100:1	0.0017	***
Fn3	HPA	1:1	Fn3	HPA	10:1	< 0.0001	***
Fn3	HPA	1:1	Fn3	HPA	100:1	< 0.0001	***
Fn3	HPA	1:10	Fn3	HPA	100:1	0.0025	***
Fn3	Gluc	1:1	Fn3	Gluc	10:1	0.5988	ns
Fn3	Gluc	1:1	Fn3	Gluc	100:1	0.0666	ns
Fn3	Gluc	1:10	Fn3	Gluc	100:1	0.0565	ns
hTF	mTEGa	1:1	Fn3	mTEGa	10:1	< 0.0001	***
hTF	mTEGa	1:1	Fn3	mTEGa	100:1	< 0.0001	***
hTF	mTEGa	1:10	Fn3	mTEGa	100:1	0.0001	***
hTF	HPA	1:1	Fn3	HPA	10:1	< 0.0001	***
hTF	HPA	1:1	Fn3	HPA	100:1	< 0.0001	***
hTF	HPA	1:10	Fn3	HPA	100:1	< 0.0001	***
hTF	Gluc	1:1	Fn3	Gluc	10:1	ND	--
hTF	Gluc	1:1	Fn3	Gluc	100:1	ND	--
hTF	Gluc	1:10	Fn3	Gluc	100:1	ND	--

[a] All polymers were functionalized with 0.8 equivalents of indicated side chain. Side chain structures and abbreviations are shown in Scheme 1.

[b] Molar ratio of polymer to protein in conjugation reaction.

[c] P-value not determined (ND) due to no conjugation observed for one or both samples.

[d] p > 0.05, not significant, ns; p < 0.05, significant, *; p < 0.01, very significant, **; p < 0.005, extremely significant, ***.

Table S2. P-values from t-tests of samples shown in Figure 3.

Sample 1			Sample 2			p-value[c]	Interpretation[d]
Protein	Side Chain[a]	% Func[b]	Protein	Side Chain[a]	% Func[b]		
Fn3	mTEGa	60	Fn3	mTEGa	80	0.0015	***
hTF	mTEGa	60	hTF	mTEGa	80	0.011	*
Fn3	HPA	60	Fn3	HPA	80	0.0001	***
hTF	HPA	60	hTF	HPA	80	0.0047	***
Fn3	Gluc	60	Fn3	Gluc	80	0.1866	ns
hTF	Gluc	60	hTF	Gluc	80	ND	--
Fn3	mTEGa	60	hTF	mTEGa	60	< 0.001	***
Fn3	mTEGa	80	hTF	mTEGa	80	0.0135	*
Fn3	HPA	60	hTF	HPA	60	0.1065	ns
Fn3	HPA	80	hTF	HPA	80	0.0001	***
Fn3	Gluc	60	hTF	Gluc	60	0.2378	ns
Fn3	Gluc	80	hTF	Gluc	80	ND	--

[a] Side chain structures and abbreviations are shown in Scheme 1.
[b] Percent of parent polymer functionalized with given side chain. Polymer:protein ratio was 10:1 for all conjugation reactions.
[c] P-value not determined (ND) due to no conjugation observed for one or both samples.
[d] $p > 0.05$, not significant, ns; $p < 0.05$, significant, *; $p < 0.01$, very significant, **; $p < 0.005$, extremely significant, ***.

Table S3. P-values from t-tests of samples shown in Figure 4.

Sample 1			Sample 2			p-value[c]	Interpretation[d]
Protein	% Func[a]	Pol:Prot[b]	Protein	% Func[a]	Pol:Prot[b]		
Fn3	20	10:1	Fn3	20	20:1	0.0007	***
Fn3	40	10:1	Fn3	40	30:1	0.023	*
Fn3	60	10:1	Fn3	60	100:1	< 0.0001	***

[a] Percent of parent polymer functionalized with glucamine.
[b] Molar ratio of polymer to protein in conjugation reaction.
[c] P-value not determined (ND) due to no conjugation observed for one or both samples.
[d] $p > 0.05$, not significant, ns; $p < 0.05$, significant, *; $p < 0.01$, very significant, **; $p < 0.005$, extremely significant, ***.

NMR Spectra

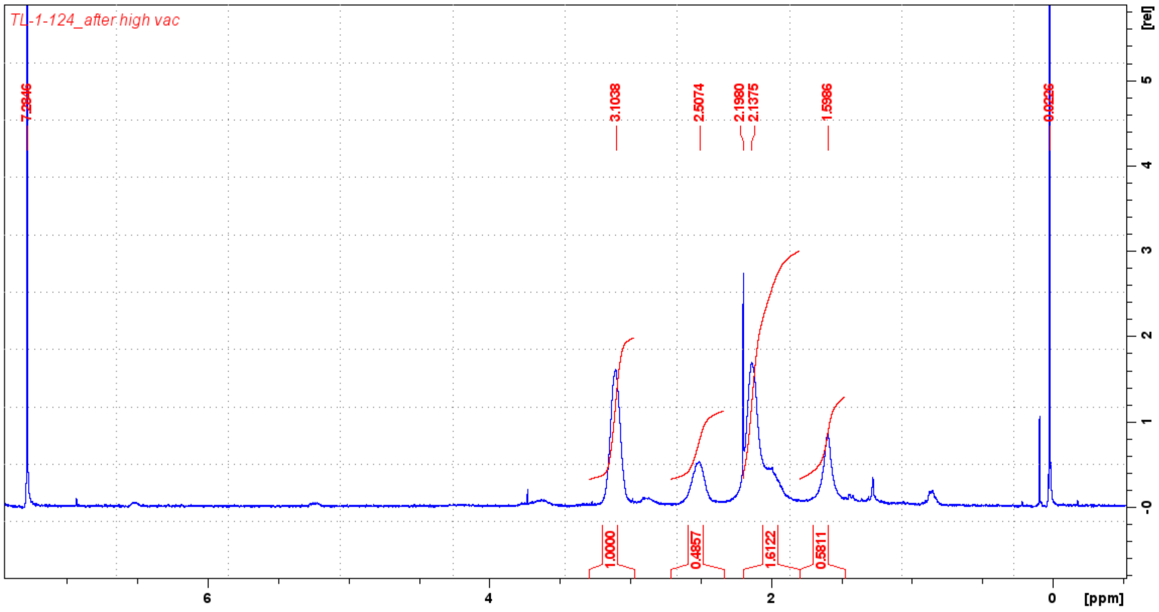

Figure S2. ^{1}H NMR spectrum of PPFPA after purification

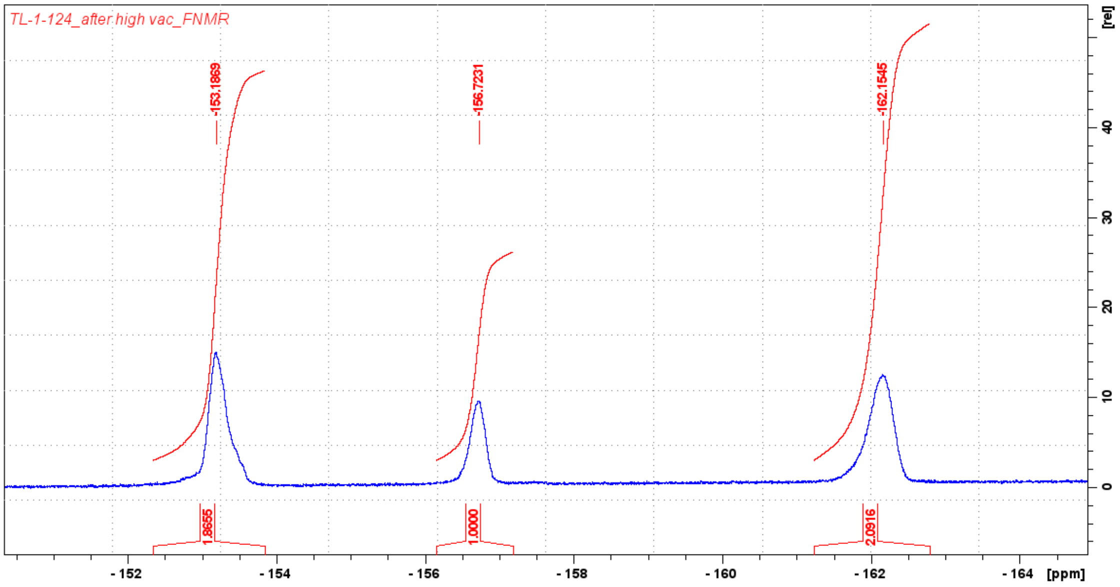

Figure S3. *^{19}F NMR spectrum of PPFPA*

Representative ^{1}H and ^{19}F NMR spectra for PPFPA-mTEGa, PPFPA-HPA, and PPFPA-Gluc polymers: All copolymers synthesized in Table 1 exhibited very similar ^{1}H and ^{19}F NMR spectra for polymers with the same side chain, but with varying grafting densities. Only the integrations varied depending on the grafting density. We have included sample ^{1}H and ^{19}F NMR spectra before and after purification for PPFPA treated with 0.4 equivalents of each of the three side chains.

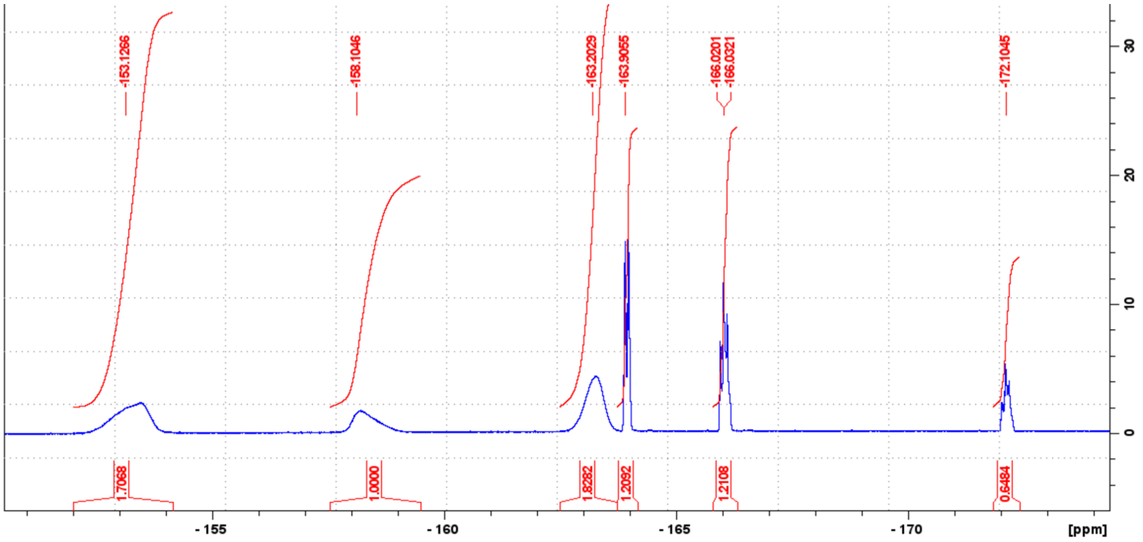

Figure S4. ^{19}F NMR spectrum of P(PFPA)$_{60}$-mTEG$_{40}$ before purification

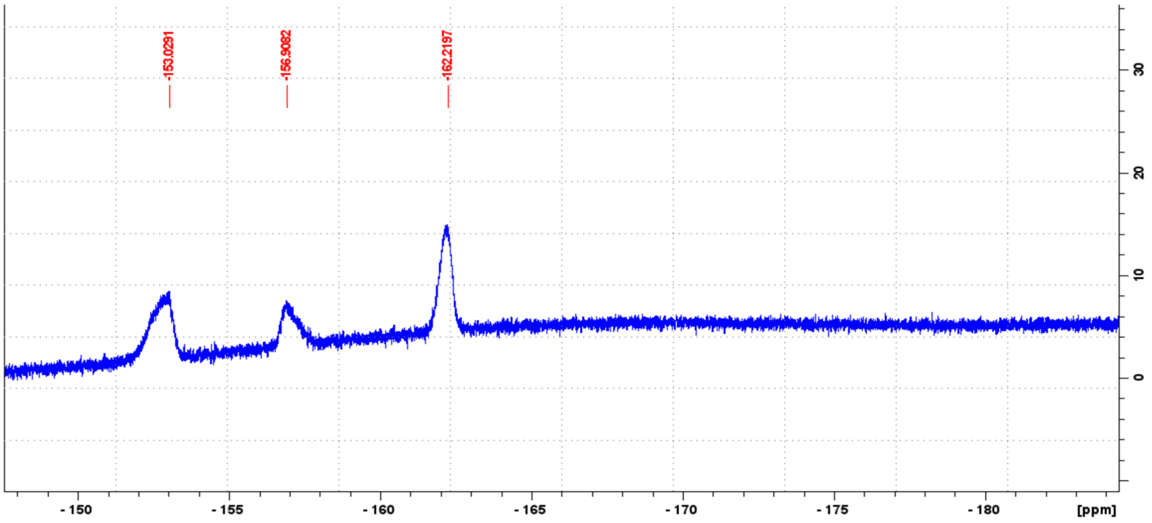

Figure S5. ^{19}F NMR spectrum of P(PFPA)$_{60}$-mTEGa$_{40}$ after purification

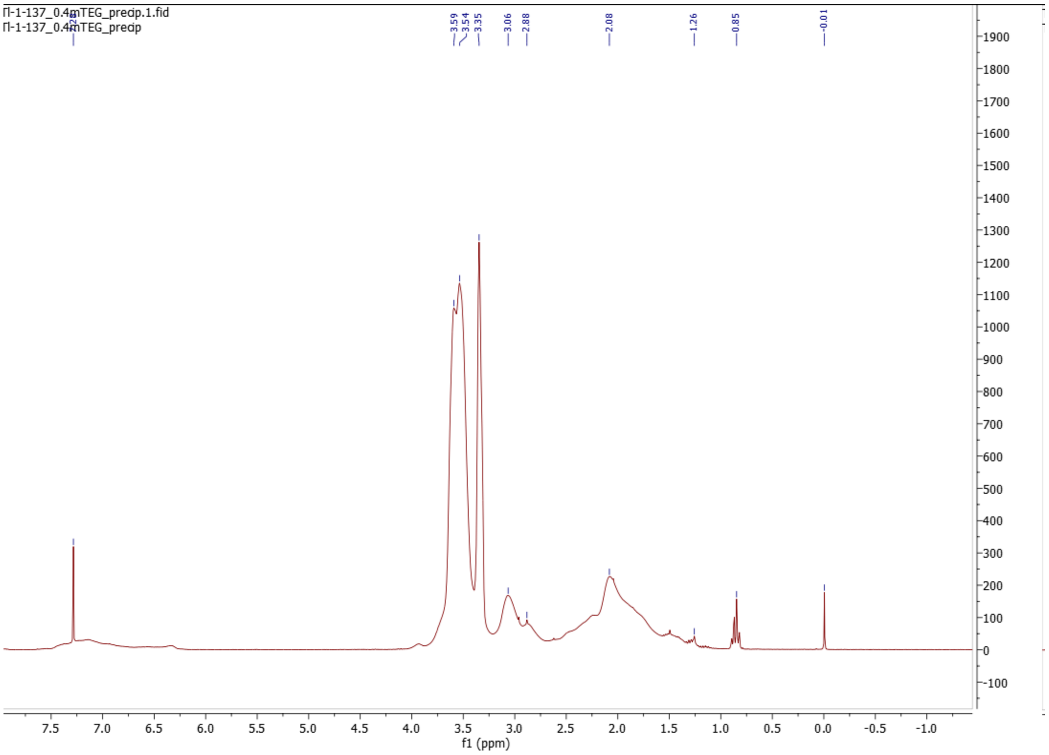

Figure S6. ^{1}H NMR spectrum of P(PFPA)$_{60}$-mTEGa$_{40}$ after purification

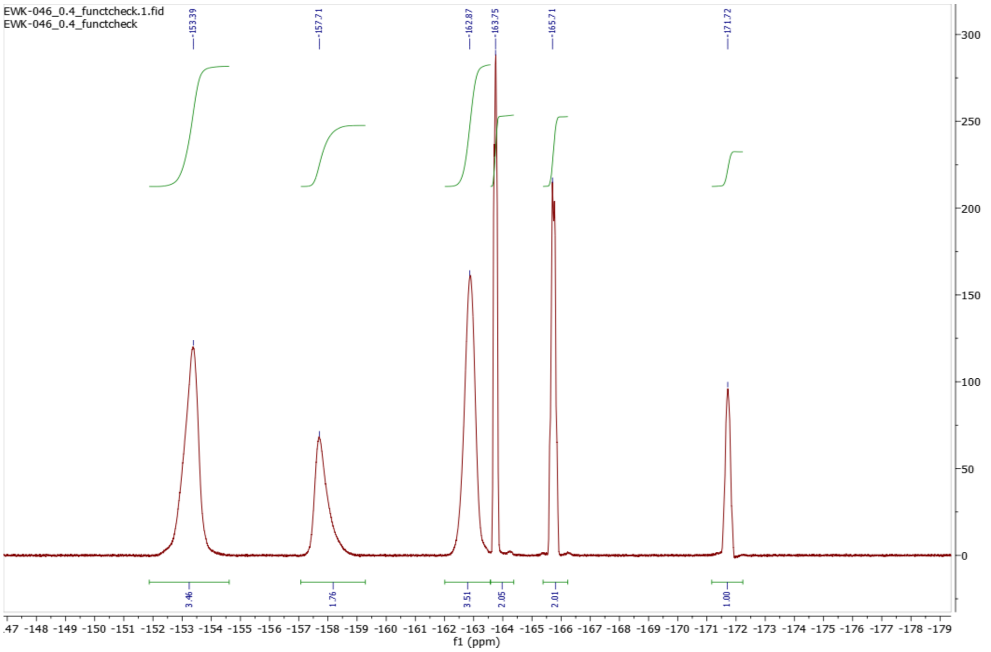

Figure S7. ^{19}F NMR spectrum of P(PFPA)$_{60}$-HPA$_{40}$ before purification

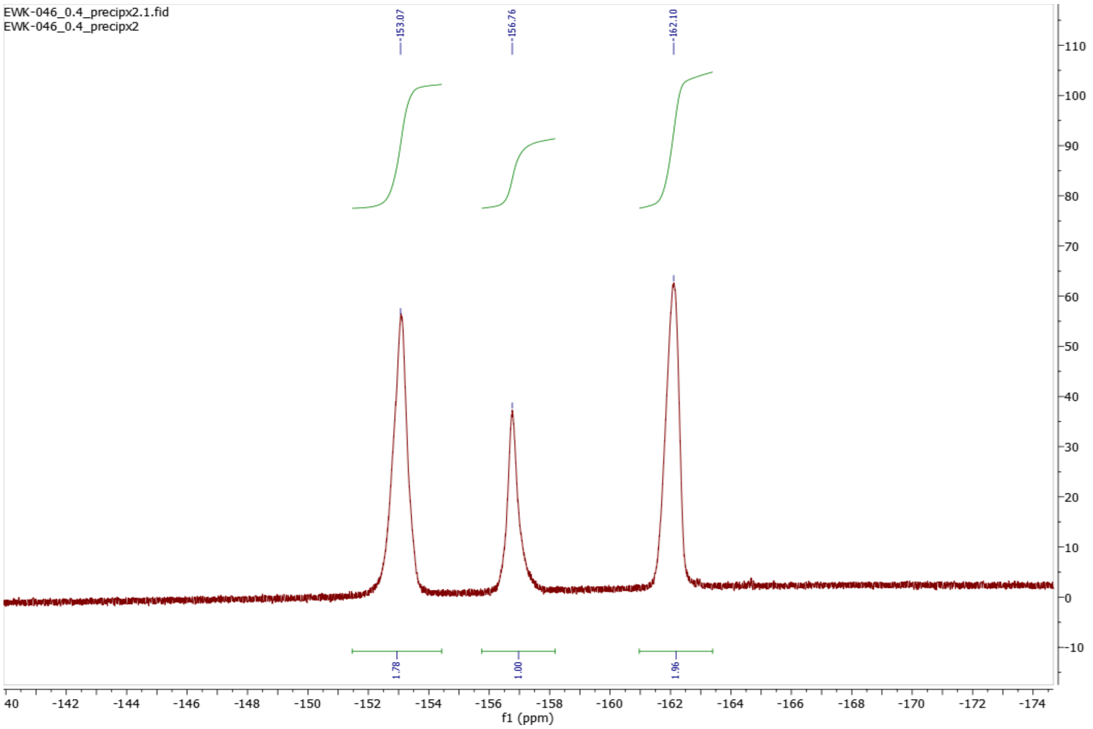

Figure S8. ^{19}F NMR spectrum of P(PFPA)$_{60}$-HPA$_{40}$ after purification

^{1}H NMR spectrum of P(PFPA)$_{60}$-HPA$_{40}$ after purification

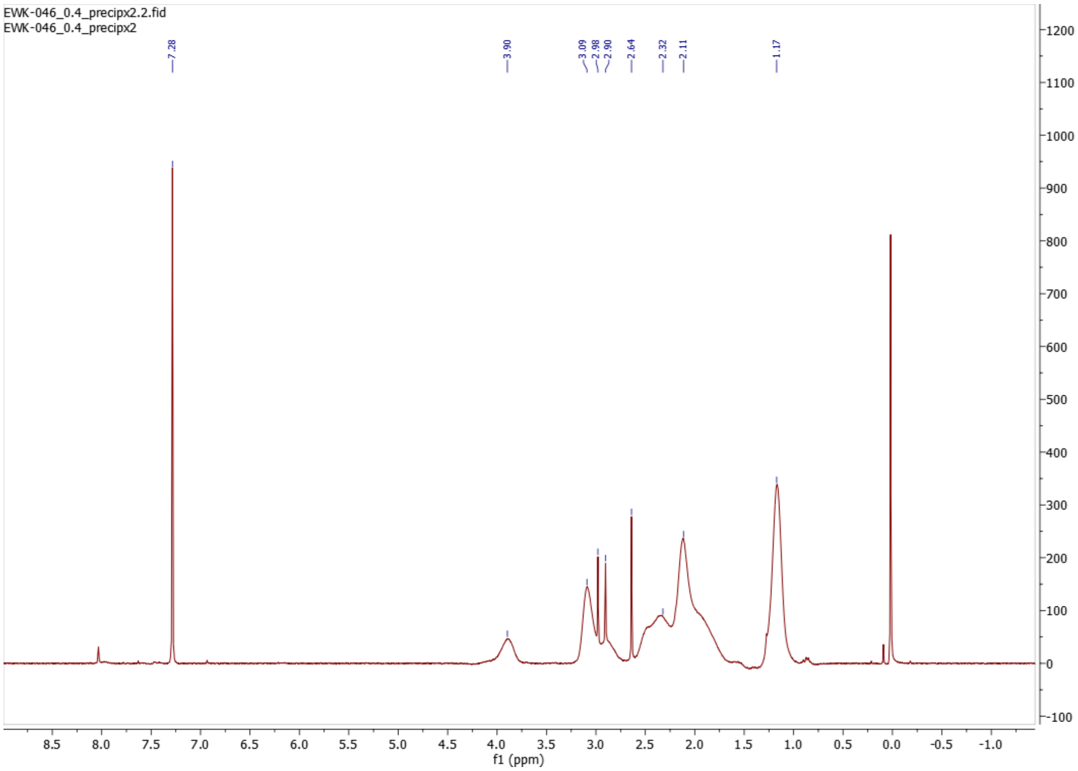

Figure S9. ^{1}H NMR spectrum of P(PFPA)$_{60}$-HPA$_{40}$ after purification

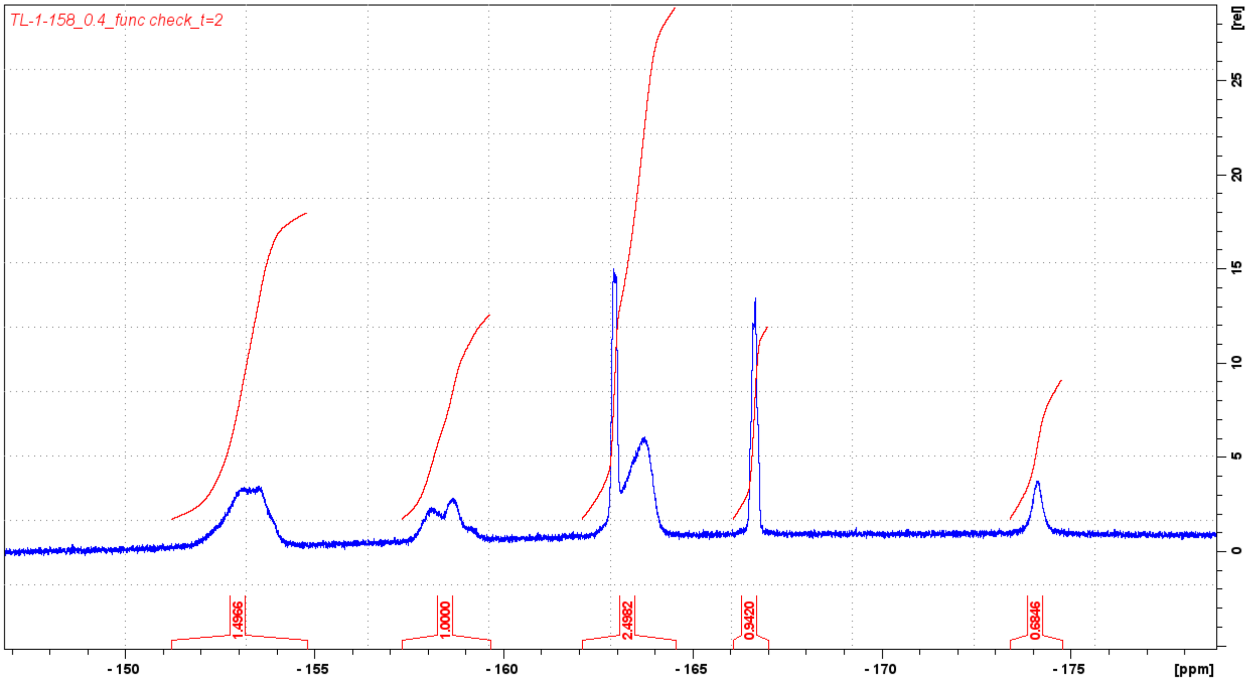

Figure S10. ^{19}F NMR spectrum of P(PFPA)$_{60}$-Gluc$_{40}$ before purification

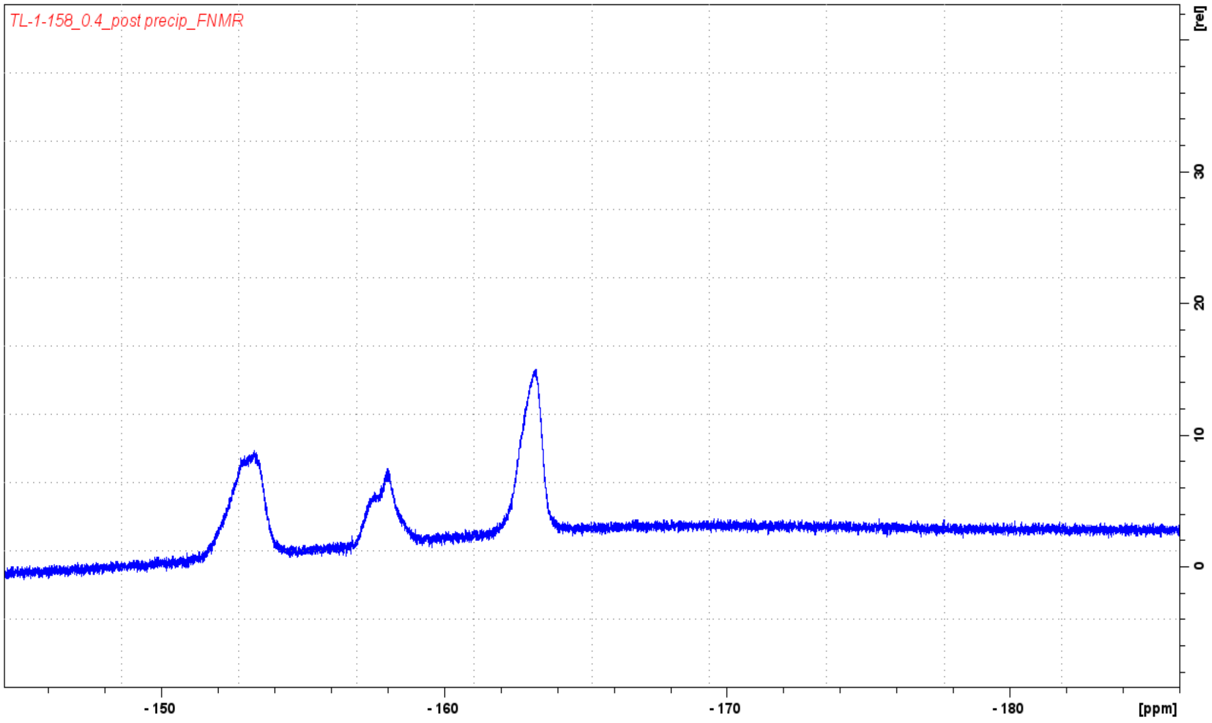

Figure S11. ^{19}F NMR spectrum of P(PFPA)$_{60}$-Gluc$_{40}$ after purification

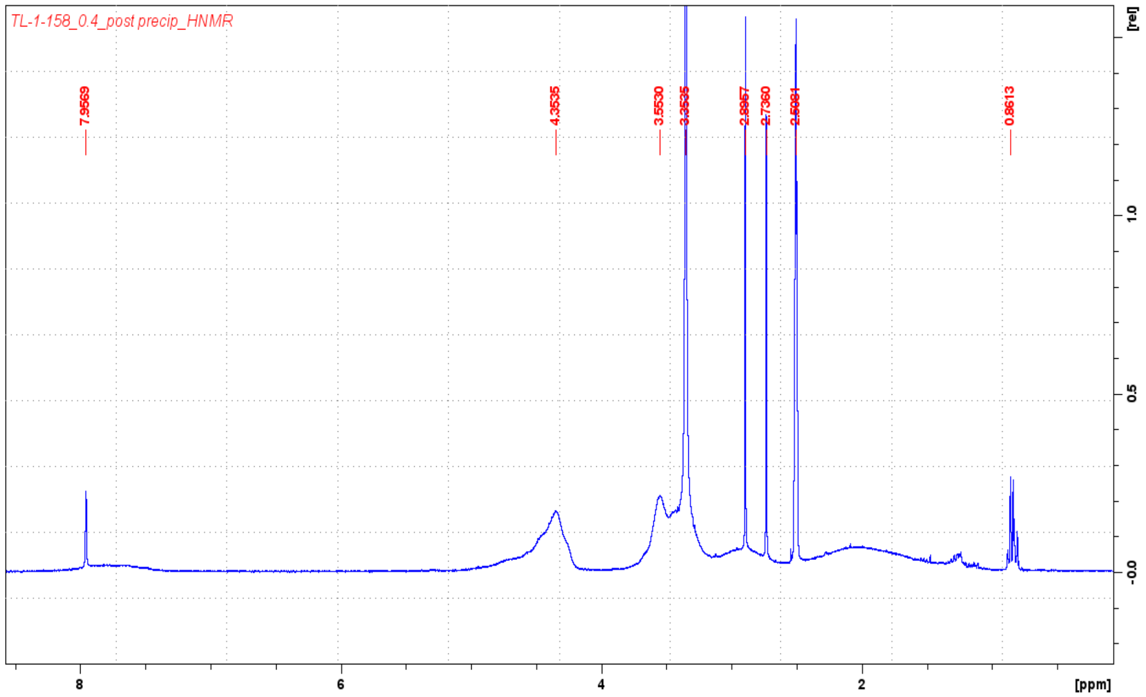

Figure S12. ^{1}H NMR spectrum of P(PFPA)$_{60}$-Gluc$_{40}$ after purification

Representative GPC traces for PPFPA, PPFPA-mTEGa, and PPFPA-HPA (co)polymers

GPC traces corresponding to the data shown in Table 1 of the main text of the paper are shown below. All GPC samples were run with a toluene standard (retention time ~12 min) to monitor flow rate.

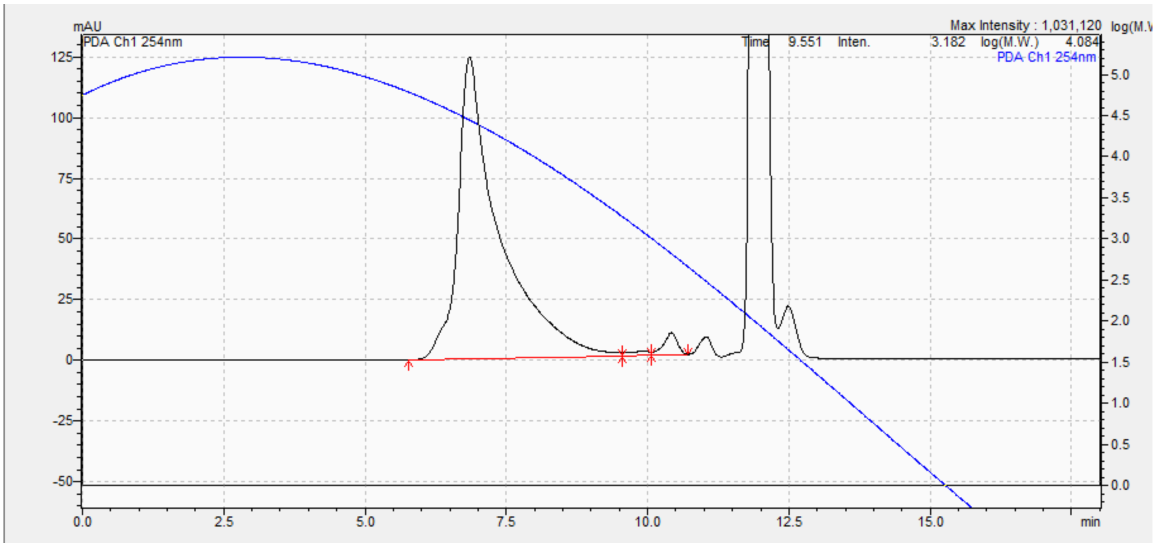

Figure S13. GPC Trace of PPFPA homopolymer

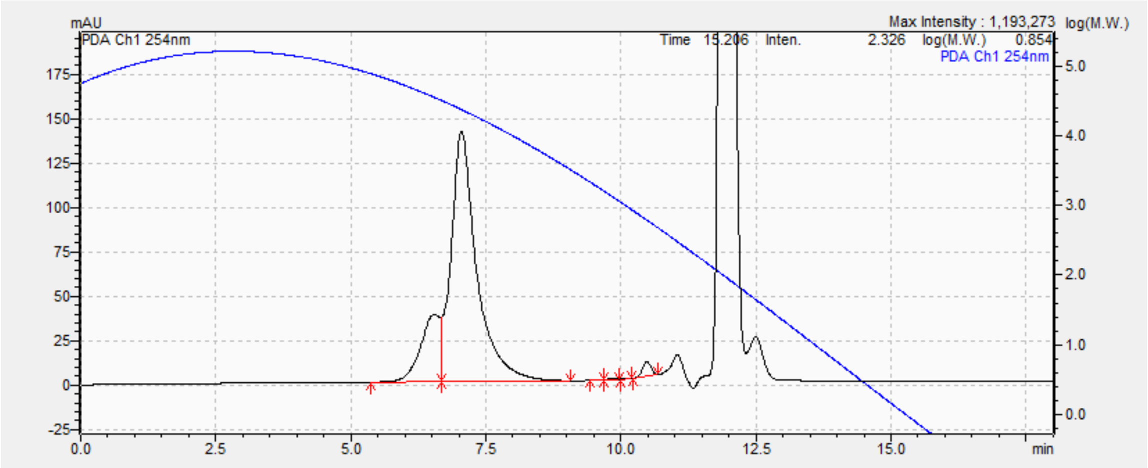

Figure S14. GPC Trace of P(PFPA)$_{80}$-mTEGa$_{20}$ copolymer

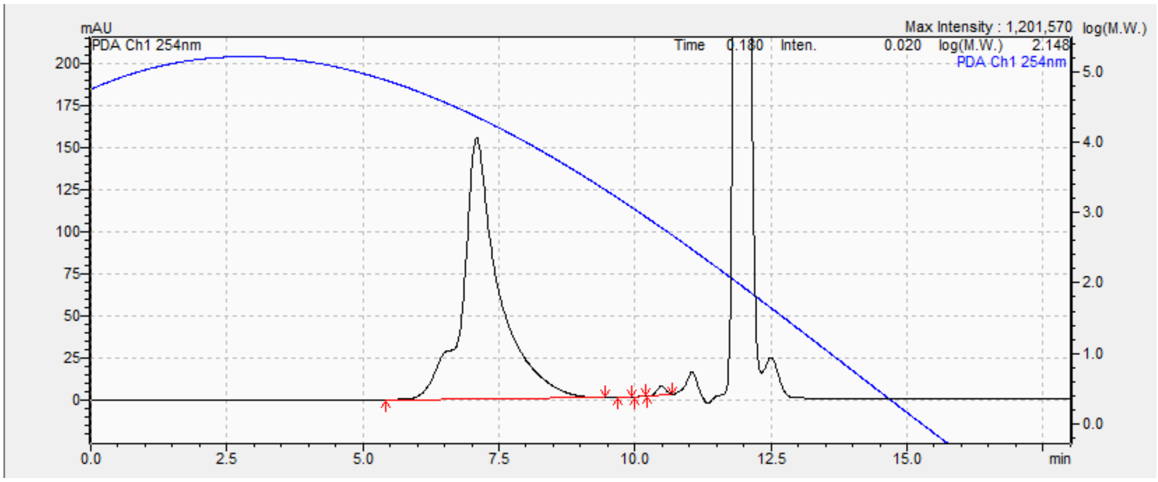

Figure S15. GPC Trace of P(PFPA)$_{60}$-mTEGa$_{40}$ copolymer

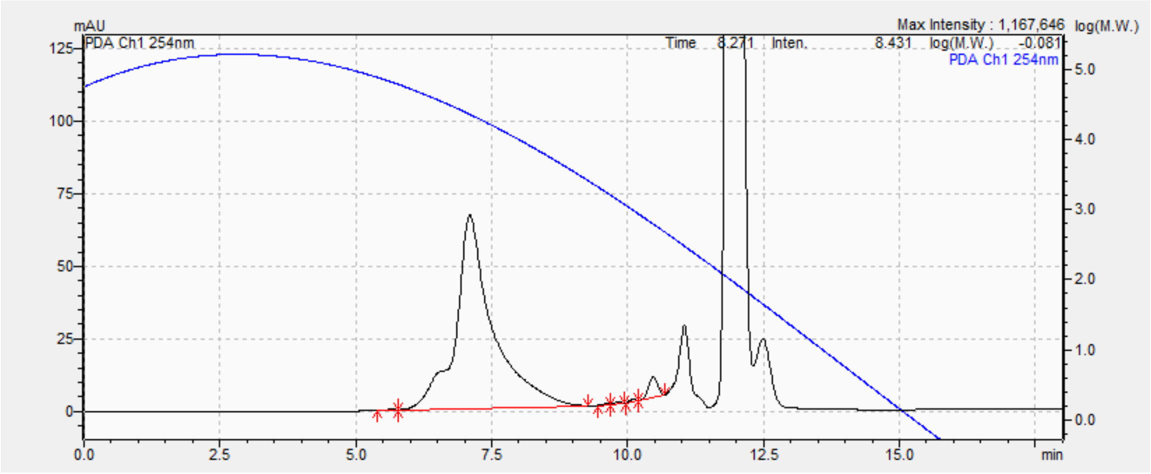

Figure S16. GPC Trace of P(PFPA)$_{40}$-mTEGa$_{60}$ copolymer

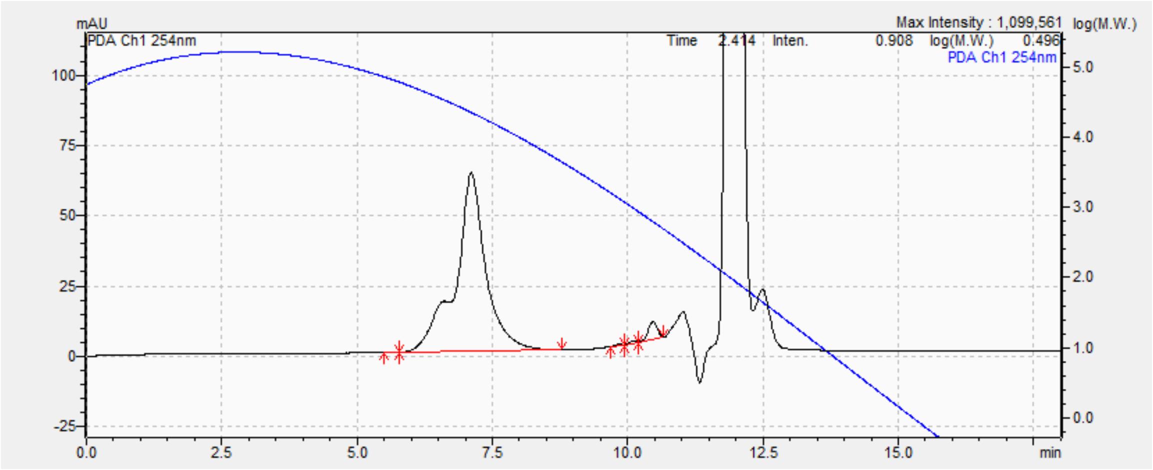

Figure S17. GPC Trace of P(PFPA)$_{20}$-mTEGa$_{80}$ copolymer

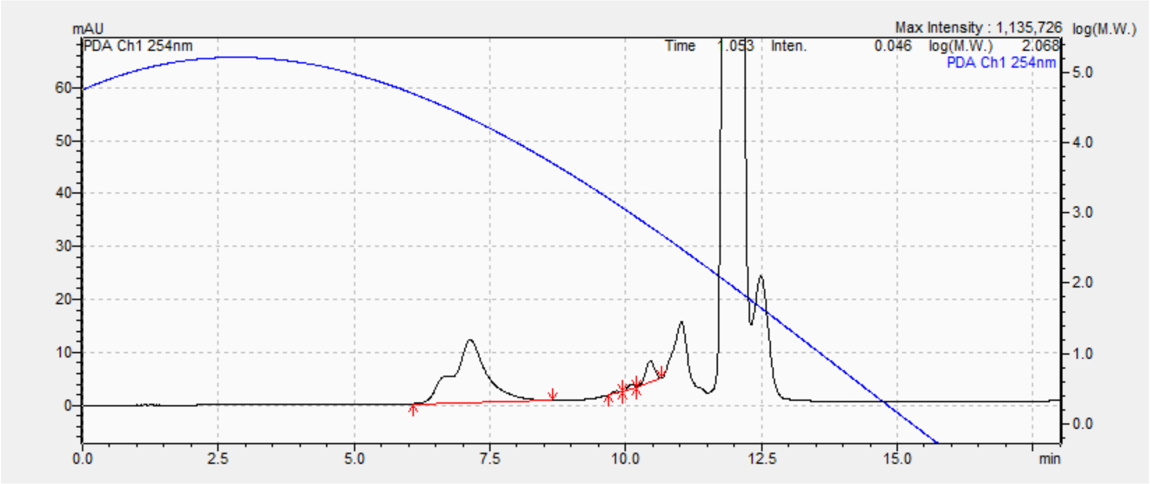

Figure S18. GPC Trace of P(PFPA)$_0$-mTEGa$_{100}$ homopolymer

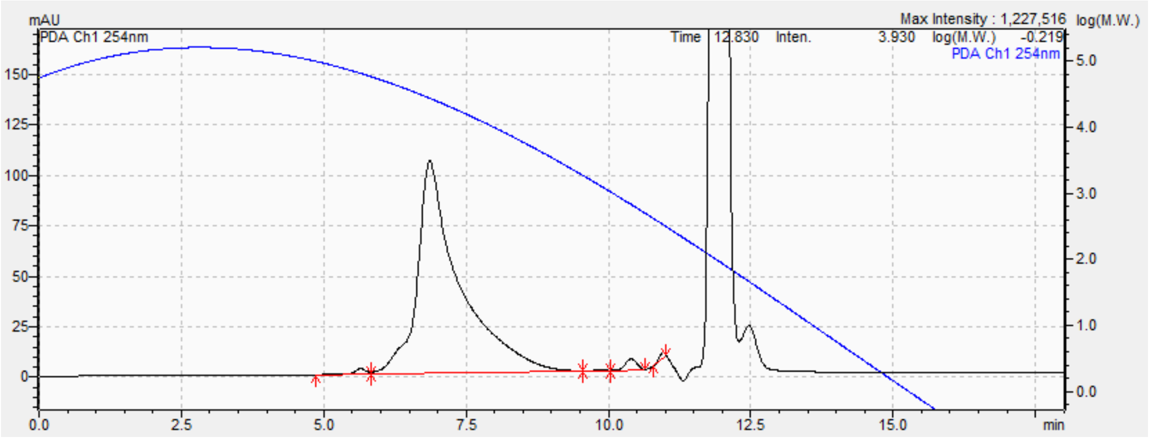

Figure S19. GPC Trace of P(PFPA)$_{80}$-HPA$_{20}$ copolymer

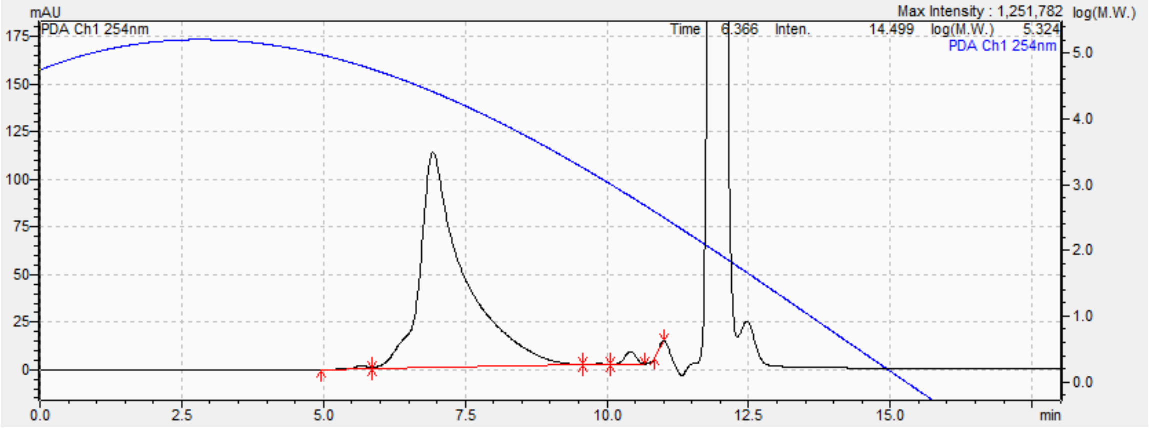

Figure S20. GPC Trace of P(PFPA)$_{60}$-HPA$_{40}$ copolymer

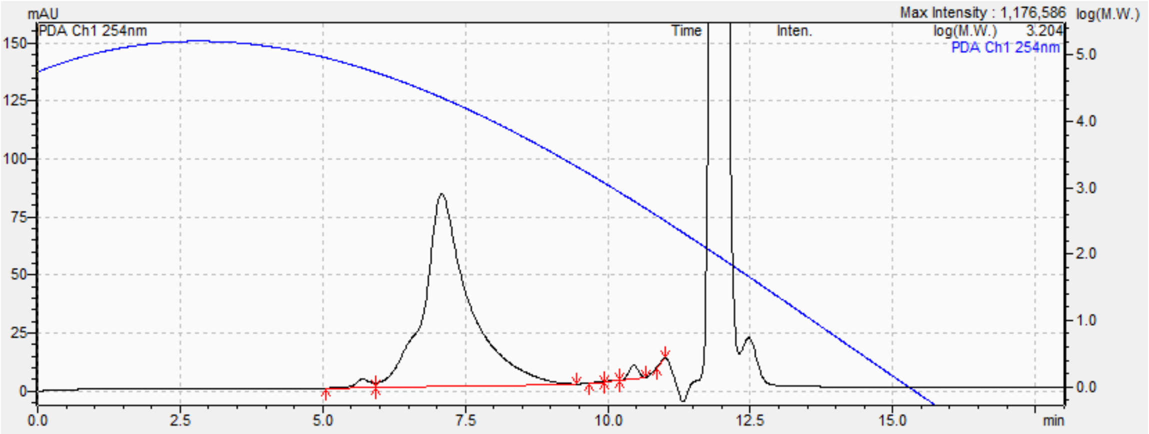

Figure S21. GPC Trace of P(PFPA)$_{40}$-HPA$_{60}$ copolymer

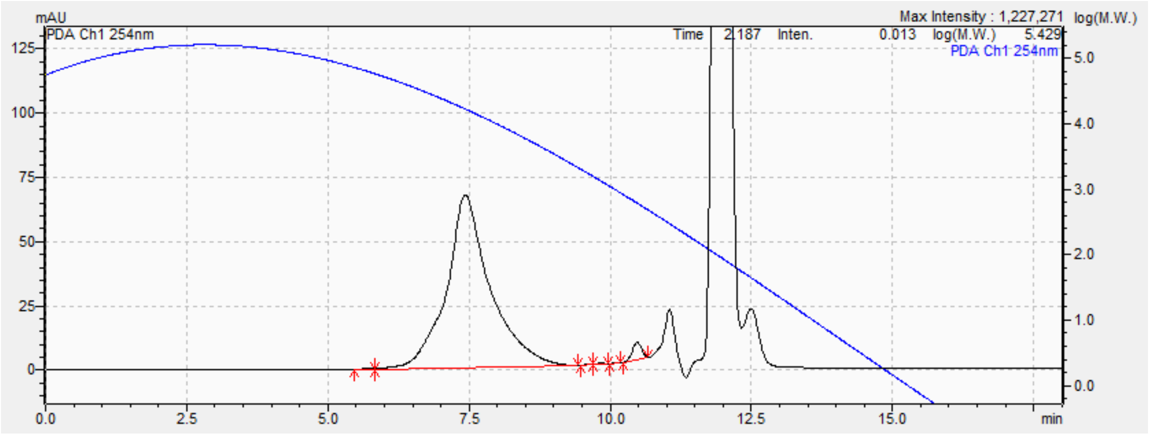

Figure S22. GPC Trace of $P(PFPA)_{20}$-HPA_{80} copolymer
